# Performance and stability of LaTiO_2_N based photoanodes at varying electrolyte temperatures and irradiances

**DOI:** 10.1039/d6el00054a

**Published:** 2026-06-24

**Authors:** Julian Hörndl, Jakub Zalesak, Franky E. Bedoya-Lora, Sophia Haussener, Simone Pokrant

**Affiliations:** a Department of Chemistry and Physics of Materials, Paris Lodron University Salzburg Jakob-Haringer-Str. 2A 5020 Salzburg Austria simone.pokrant@plus.ac.at; b Laboratory of Renewable Energy Science and Engineering, Ecole Polytechnique Federale de Lausanne 1015 Lausanne Switzerland

## Abstract

Operating conditions greatly affect the performance and lifetime of photoelectrochemical systems. Understanding these influences is required for the development of photoelectrochemical water splitting towards a practical and scalable solution for solar hydrogen generation. This study investigates the influence of two important operating parameters (*i.e.* irradiance and electrolyte temperature) on the efficiency and stability of LaTiO_2_N-based photoanodes with NiO_*x*_ and CoO_*x*_ cocatalysts as representative examples for oxynitride electrodes. Chronoamperometry measurements at 1.23 V *vs.* RHE are performed exploring a wide range of irradiances (1000–119 000 W m^−2^) and electrolyte temperatures (17–50 °C). An increase in the electrolyte temperature leads to a decrease in the photoanode efficiency by 52% and in the stability by 8% respectively, while higher irradiances improve initial efficiencies up to 77% but decrease the stability of the photoanodes by 38%. The most suitable operating point is obtained at a 1000 W m^−2^ irradiance and an electrolyte temperature of 17 °C. Further analysis using XRD, SEM, STEM-EXD/EELS, HREM, and ICP-MS revealed that degradation is mostly driven by a cocatalyst dissolution/redeposition mechanism, which is accelerated by increased electrolyte temperatures and especially irradiances, while bulk LaTiO_2_N remained stable apart from surface oxidation.

## Abstract 2

Low electrolyte temperatures and irradiances are favourable for the efficiency and stability of LaTiO_2_N-based photoanodes. The main degradation cause is cocatalyst dissolution/redeposition, while bulk LaTiO_2_N is stable except for surface oxidation.

## Graphical abstract

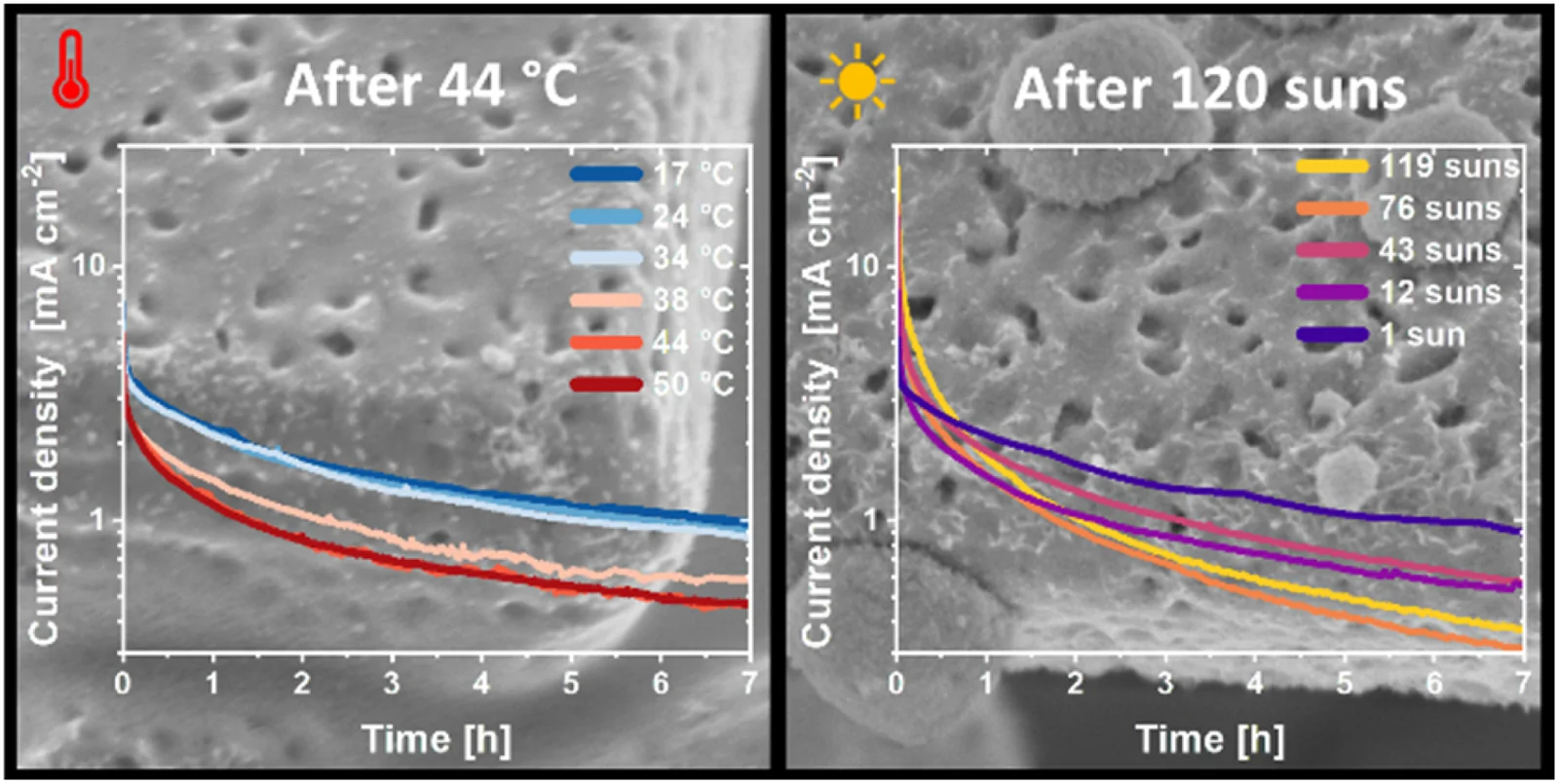

**Broader context**

The global transition to sustainable energy sources is one of the most pressing challenges of our time. Solar fuels, specifically solar hydrogen, have emerged as a promising solution to store and transport renewable energy. Photoelectrochemical water splitting, a technology that converts sunlight directly into hydrogen, offers a clean and scalable pathway to decarbonize our energy system. However, the efficiency and stability of photoelectrochemical systems are highly dependent on operating conditions, such as irradiance and electrolyte temperature. Previous studies on oxide-based photoanodes, such as BiVO_4_ and Fe_2_O_3_, have shown that these interdependencies are strongly material-dependent. To the best of our knowledge, this study presents the first dedicated investigation into the influence of operating conditions on the performance and stability of LaTiO_2_N-based photoelectrodes, a material class that is representative of perovskite-related oxynitrides. This material class has shown record performance as a photoanode for the challenging oxygen evolution reaction. While higher irradiance initially improves efficiency, it also accelerates degradation, whereas increasing electrolyte temperature reduces both efficiency and stability. Identifying cocatalyst dissolution as the primary degradation mechanism—while LaTiO_2_N remains stable apart from surface oxidation—provides key insights for the development of more robust solar hydrogen production systems.

## 1 Introduction

Harvesting energy from renewable and carbon-free sources to produce chemical energy, such as the solar fuel hydrogen, holds great promise for addressing future energy demands.^2–5^ Thanks to its less complex setup, photoelectrochemical (PEC) water splitting offers a cost-effective alternative to the combination of photovoltaic cells and electrolysers.^6–8^ A tandem PEC system comprises two electrodes that are physically separated but electrically connected through an ohmic contact, where the photocathode facilitates the hydrogen evolution reaction (HER), while the photoanode drives the oxygen evolution reaction (OER).^10,11^ For assessing the performance of a PEC system, the solar-to-hydrogen (STH) efficiency is widely recognized as a benchmark metric.^10,12^ It represents the ratio of the chemical energy contained in the produced hydrogen to the incident solar energy during the operating time of the system.^10^

For PEC systems to be viable for large-scale deployment, they must meet four critical criteria: high efficiency, long-term stability, scalability, and environmental sustainability.^3,6,10^ One way to define the target value for each parameter is technoeconomic studies, which focus on the economic viability of the PEC system.^3^ Recent studies have proposed various combinations of target values for efficiency, lifetime, and scalability; however, the suggested values differ significantly across publications.^3,6,12^ For example, Schneidewind *et al.* proposed a STH of *E*%, a lifetime of two years and manufacturing costs below 300 $ per m^2^, while Segev suggested a STH of 10%, a lifetime of ten years and manufacturing cost below 1500 $ per m^2^.^3,6^. This implies that a STH of 10% is sufficient for PEC systems with ten years of lifetime and production costs below 1500 $ per m^2^, but not for PEC systems with two years of lifetime and production costs of 300 $ per m^2^. These examples show that the respective parameters are interrelated, as they all contribute to the levelized cost of hydrogen for the system (*i.e.* the ratio of the operating and construction costs to the amount of produced hydrogen).^13^ Therefore, it is not meaningful to fix target values for efficiency, stability or scalability alone without specifying the other two. While technoeconomic studies evaluate the economic viability of a system, life cycle assessments focus on the total energy balance as an indicator *i.e.* the ratio between the energy output in the form of green hydrogen and the primary energy consumption during fabrication.^14^ For large-scale viability, this ratio must exceed one.^15^ However, according to a recent study by Tam *et al.* state-of-the-art PEC systems still fall significantly short of this target even in the most optimistic scenario.^16^ Meanwhile for PV-EC values up to 3.7 are possible in the best-case scenario.^16^ To close this gap the most relevant parameter for PEC is the total energy output during its lifetime, *i.e.* again a combination of stability and efficiency, rather than just one of them.^14^ Therefore, it is reasonable to use the energy output as a target value, before additional indicators such as economic criteria are considered. When considering the energy balance of a specific PEC device, the energy output depends on the energy consumed for fabricating its components while the generated energy is determined by the choice of the material system, for example, photocatalysts, cocatalysts or the electrolyte composition, and on the selected operating conditions. Therefore, developing PEC systems in view of potential upscaling requires not only research in photoelectrode and device design, but also studies related to operating conditions to maximize total energy conversion (*i.e.* the combination of efficiency and lifetime).^15,17^ Relevant operating conditions for PEC devices include the electrolyte temperature and the irradiance.^8,17,18^

Regarding the electrolyte temperature dependence of PEC performance, a few studies investigated the impact of the electrolyte temperature on efficiency and stability of different types of photoelectrodes. For instance in 2014, Dias *et al.* demonstrated that increasing the electrolyte temperature enhanced the photocurrent density of Si-doped Fe_2_O_3_ based photoanodes.^19^ Twelve years later the same effect was observed by Liu *et al.* on CuWO_3_ based photoanodes and electrolyte temperatures up to 60 °C.^20^ In both cases, the increased photocurrent density was attributed to an increase in the charge carrier density with an increase in the electrolyte temperature.^19,20^ Meanwhile the opposite effect was observed by Valenza *et al.* on Fe_2_O_3_ based photoanodes,^21^ where the decreased photocurrent density at elevated electrolyte temperatures was attributed to an increase in the charge carrier recombination rate.^21^ Hence, the beneficial effect of the increased charge carrier density was outweighed by the detrimental effect of the increased charge carrier recombination rate. These results show that the impact of the electrolyte temperature on the photoanode's performance is also strongly influenced by changes in the charge transport properties with temperature. With respect to the influence of electrolyte temperature on electrode stability, both Holmes-Gentle *et al.* and Dias *et al.* observed a decrease in photoanode stability with increasing electrolyte temperature for BiVO_4_ and Fe_2_O_3_ based photoanodes.^19,22^ In both cases the decrease in stability was attributed to an acceleration of degradation processes.^19,22^ These studies show that the effect of electrolyte temperature on PEC performance is significant and that its impact is material dependent, demonstrating the need for further investigations of other relevant material classes. In addition to the direct influence of the electrolyte temperature on photoelectrode performance temperature extremes pose additional risks for an application in the field. Electrolyte freezing can halt device operation, while overheating may degrade components and reduce stability.^23^ In both scenarios a temperature-controlled water bath offers a practical solution to maintain optimal operating conditions.^23^

Regarding the influence of another important operating parameter, *i.e.* the irradiance, on photoanode efficiency, studies by Wang *et al.* in 2008 on GaInP_2_ based photoanodes under concentrated illumination (10 kW m^−2^) revealed a non-linear increase in the measured current with increasing irradiance.^24^ A study by Villanova *et al.* in 2020 on Fe_2_O_3_ based photoanodes at irradiance up to 13 kW m^−2^ reported a logarithmic correlation between the photocurrent and the irradiance.^25^ This observation was attributed to ohmic losses caused by increased charge carrier recombination and insufficient charge carrier transfer between the semiconductor and the current collector.^25^ After correction for ohmic losses Segev *et al.* and Gupta *et al.* observed a linear correlation between the irradiance and the photocurrent for Fe_2_O_3_ and LaFeO_3_ based photoelectrodes under irradiances up to 25 kW m^−2^.^26,27^ The first investigation of the long-term stability under high irradiances by Villanova *et al.* in 2020 on Fe_2_O_3_ based photoanodes showed a relatively stable photocurrent of around 2 mA cm^−2^ for 13.5 h of operation using an irradiance of around 12 kW m^2^. However, no systematic investigations regarding the influence of irradiance on stability were made. A more sophisticated and systematic study on this topic was reported by Holmes-Gentle and Bedoya *et al.* in 2023 on BiVO_4_ based photoanodes. By performing chronoamperometry at 1.23 V *vs.* RHE under irradiances ranging from 1 kW m^2^ to 358 kW m^−2^, they observed higher initial currents but also noticeably faster current decays at higher irradiances.^22^ These results emphasize the importance of conducting systematic studies over an irradiance range on stability and efficiency of a photoanode.

As demonstrated in the literature, both irradiance and electrolyte temperature significantly influence photoanode efficiency and stability. It was further discussed that especially the influence of the electrolyte temperature on photoanode efficiency is strongly dependent on the photoelectrode architecture (*i.e.* electrode use, photoactive material(s), dopant(s), material topography, surface cocatalyst(s), *etc.*). These results emphasize the importance of comprehensive studies regarding the influence of irradiance and electrolyte temperature on photoanode efficiency and stability across different types of materials and photoelectrode designs. To date, most research in this area has focused on oxides such as BiVO_4_ or Fe_2_O_3_.^19,21,22,28^ Based on these studies, degradation mechanisms and respective strategies for their mitigation have been proposed for those materials. Meanwhile (oxy)nitrides, such as TaON, Ta_3_N_5_, GaN or BaTiO_2_N, have received comparatively little attention, even though they have shown promising performance regarding both efficiency and stability.^29–33^ Another prominent example is the oxynitride LaTiO_2_N (LTON), which is already well studied in terms of efficiency and has shown competitive photoelectrochemical performance (2.5–8.9 mA cm^−2^ at 1.23 V *vs.* RHE^29,34,35^). Meanwhile its stability and the relevant degradation mechanisms have not been studied intensively until we recently reported its degradation under standard operating conditions (*i.e.* 1 sun irradiance and room temperature).^36^ By conducting a detailed analysis of the chronoamperometric curve progression of oxynitride based photoanodes, it was found that all investigated chronoamperometry curves showed a sharp initial decay within the first minutes followed by a more gradual decline over the remaining measuring time and could therefore be described by the sum of two exponential terms.^36^ Here, the first exponential term was attributed to a transient capacitive current which can be disregarded when evaluating long-term stability. Meanwhile the second term corresponded to the photogenerated current, offering a more reliable estimation of the generated photocurrent at *t* = 0, I_0,pc_, and the photoanode stability.^36^ The characterisation of LTON-based photoanodes after chronoamperometry, under one sun illumination and without temperature control, revealed a performance loss caused by the combination of surface oxidation of the LTON particles and the loss of cocatalysts due to dissolution.^36^ However, it remained unclear whether the selected operating conditions were suitable for achieving high energy output or improved longevity. Therefore, it is important to conduct a systematic study on stability and performance of LTON as a function of electrolyte temperature and irradiance to identify the most suitable operating point for this type of photoanode.

In this study the influence of the irradiance and the electrolyte temperature on the efficiency and stability of LTON-based photoanodes was assessed by performing chronoamperometry at 1.23 V *vs.* RHE under irradiances between 1 sun (≈1000 W m^−2^) and 119 suns and at electrolyte temperatures between 17 °C and 50 °C. The optimal operating conditions were then determined by comparing the efficiency and stability of the LTON photoanodes within the assessed range of temperature and irradiance. To gain further insight into the mechanisms behind the influence of temperature and irradiance on stability, LTON-based photoanodes were characterized before and after seven-hour chronoamperometry using SEM, XRD, HREM and STEM-EDX/EELS, to identify any morphological, compositional or structural changes compared to pristine photoanodes. The electrolyte composition was also analysed using ICP-MS before and after chronoamperometry. To further elucidate the temperature dependency, the oxygen evolution rates of LTON particles at temperatures ranging from 10 to 40 °C were determined and compared to the photoanode performance. In addition, electrochemical impedance spectroscopy (EIS) was used to investigate the influence of temperature on the charge transfer efficiency of the photoanodes. Based on these results, the most suitable operating point for the LTON-based photoanodes within the investigated range was obtained for an irradiance of 1 sun and an electrolyte temperature of 17 °C.

## 2 Experimental section

### 2.1 Materials and electrodes

Following the synthesis described by Werner *et al.* LTON was prepared *via* a two-step process.^37^ For the synthesis of the precursor La_2_Ti_2_O_7_ (LTO), 6.25 mmol La_2_O_3_ (Sigma-Aldrich, 99,99%), 12.5 mmol TiO_2_ (Aldrich, >99%, Anatase) and 1.25 mol NaCl (VWR, 99%) were mixed on a roll mill together with isopropanol and zirconia balls. The resulting mixture was washed with isopropanol and dried at 70 °C for 12 hours. The dried powder was heated at a rate of 10 K min^−1^ to 500 °C and then at 1 K min^−1^ to 1200 °C, where it was held for 10 hours before cooling to room temperature. The product was washed with 5 L deionized water and dried at 100 °C for 10 hours. To convert LTO to LTON, the LTO was thermally treated at 950 °C under a 0.2 L min^−1^ NH_3_ flow for 18 hours and then cooled to room temperature under NH_3_ flow.

The electrode fabrication process followed the procedure established by Landsmann *et al.*^38^ 2.5 × 1.25 cm^2^ fluorine-doped tin oxide (FTO) substrates (Solaronix, TCO15A) were cleaned using ultrasonication in a sequence of solutions: 2% Hellmanex aqueous solution, deionized water, and acetone. After cleaning, the substrates were stored in isopropanol (Sigma-Aldrich, p.a.). For the dispersion preparation, 43.5 mg LTON and 12.5 mg iodine were mixed in 62 mL of acetone (Sigma Aldrich, p.a.) and subjected to 10 minutes of ultrasonication. After resting for at least 1 hour, the dispersion was ultrasonicated again for another 10 minutes before use. During the deposition process, two FTO substrates were immersed in the dispersion, separated by a polytetrafluoroethylene (PTFE) spacer with a thickness of 6.5 mm. A voltage of 20 V was applied between the substrates for 3.5 minutes, with the dispersion stirred at 450 rpm for 10 seconds at the end of each minute. After each deposition, the dispersion was ultrasonicated for 2 minutes. A total of eight photoanodes were fabricated using the same dispersion. Post-deposition treatments were conducted based on methods described in ref. 34. The electrodes were first immersed in a 0.2 M ethanolic solution of TiCl_4_ for 10 seconds and then left to dry for 1 hour at room temperature. Following this, they were annealed at 370 °C for 30 minutes under a 0.1 L min^−1^ flow of NH_3_ gas. Next, the electrodes were dipped in a 0.2 M ethanolic solution of TaCl_5_ for 10 seconds, dried under ambient conditions for 1 hour, and annealed under the same conditions as those used after the TiCl_4_ treatment (370 °C for 30 minutes with a 0.1 L min^−1^ NH_3_ flow). For the cocatalyst deposition, the electrodes were immersed in a 0.05 M ethanolic solution of Ni(NO_3_)_2_ (>99%, Millipore) and Co(NO_3_)_2_ (>99.5%, Millipore) for 20 seconds, dried at room temperature for 1 hour, and annealed in air for 10 min at 200 °C and 150 °C, respectively.

### 2.2 X-ray diffraction

For powder X-ray diffraction (XRD) a Bruker D8 Advance diffractometer with a goniometer radius of 280 mm and a fast solid-state LynxEye detector was used. The different LTO and LTON powders were mixed with isopropanol and then drop cast onto a thin zero-background single-crystal silicon sample holder. The measurements were carried out using Cu Kα_1_ (1.5406 Å) and Kα_2_ (1.5444 Å) radiation in a 2 : 1 ratio, with 2*θ* ranging from 5° to 95° at a step size of 0.02°. For investigation of the phase purity of the synthesized LTO and LTON powders, the obtained diffractograms were then compared with corresponding references from the ICSD.

### 2.3 Scanning electron microscopy

A Zeiss Ultra Plus 55 scanning electron microscope (SEM), equipped with an in-lens secondary electron detector, was used to analyse the morphology of the LTON powders and LTON-based photoanodes. Imaging was performed with an accelerating voltage of 5 kV and a working distance of 4 mm. SEM images were acquired at magnifications of 25,000×, 50,000×, and 200,000×. Prior to the measurements, the powders and photoanodes were sputter-coated with gold for 120 seconds at 40 mA using a Cressington Sputter Coater 108 Auto.

### 2.4 Transmission electron microscopy

A Thermo Fisher Scientific Helios 5 FX dual beam microscope was used for the preparation of the TEM lamellae. Areas of interest were covered with electron assisted C-deposition, followed by ion assisted C-deposition layers. Chunks of LTON material were cut along the long axes of the LTON particles and transferred to carbon grids. For the thinning process accelerating voltages of 2 kV to 30 kV were used, with beam currents ranging from 25 pA to 2 nA.

Transmission electron microscopy (TEM) was performed with a JEOL-JEM-F200 TEM equipped with a cold field emission gun and a CEOS CEFID energy filter. The acceleration voltage of the TEM was set to 200 kV in both TEM and scanning TEM (STEM) modes. STEM energy-dispersive X-ray spectroscopy (EDX) elemental mapping was performed using a Thermo Fisher Scientific Titan Themis. The microscope was operated at an accelerating voltage of 300 kV and a beam current of ≈0.7 nA.

### 2.5 Induced coupled plasma mass spectroscopy

An Agilent 8900 triple quadrupole ICP-MS was used to determine the concentrations of La, Ti, Ta, Co, and Ni in the electrolyte. Electrolyte samples of 15 mL were collected both before and after the seven-hour chronoamperometry. These samples were diluted tenfold with 1% HNO_3_ to dissolve any potential precipitates. The ICP-MS measurements were conducted in helium mode.

Regarding the Ni cations, the concentrations detected in the blank samples were higher than those measured after the chronoamperometry. Further investigations revealed that the elevated Ni concentration in the blanks was due to the insufficient purity of the Na_2_SO_4_ and NaOH used for the preparation of the electrolyte. As a result of this contamination in the pristine electrolyte, it was not possible to draw any reliable conclusions about the potential dissolution of Ni ions during chronoamperometry. Similarly, the blank for the 50 °C Ta measurement was also contaminated.

### 2.6 Photocatalytic measurements

For the determination of the photocatalytic O2 evolution rate, we used a closed chamber with an argon atmosphere, partially filled with an LTON particle dispersion. For this purpose, 20 mg LTON particles were dispersed in 40 mL 20 mM AgNO_3_*via* ultrasonication for 5 minutes. Prior to each measurement the dispersion was degassed by evacuation followed by a purging of the chamber with argon. For illumination of the dispersion, a 300 W Xenon lamp was used with its irradiance adjusted to 1000 W m^−2^ and equipped with an AM 1.5 G filter. For the determination of the O_2_ evolution rate, the molar fractions of O_2_ were measured every 10 minutes for an hour using gas chromatography (type micro-GC fusion, INFICON) with the ability to detect molecular hydrogen, oxygen, and nitrogen. The chamber temperature was controlled during the measurements by immersing it in a tempered water bath. Based on the gas volume inside the chamber *V*_C_, the pressure *P*, the temperature *T* and the universal gas constant *R* in combination with the measured oxygen molar fractions *c*_O_2__ the total amount of evolved oxygen *n*_O_2__ can be calculated (eqn (1)).1
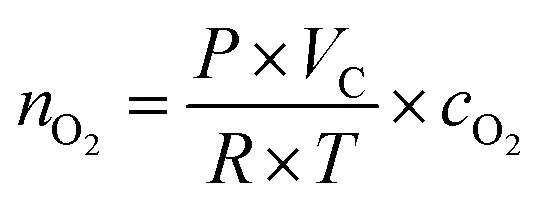


### 2.7 Photoelectrochemical measurements

To conduct simultaneous photoelectrochemical (PEC) measurements at varying irradiances and temperatures, we utilized a custom four-cell array. This setup is adapted from a design we previously reported.^39^ The PEC cells were mounted on a plexiglass fixture and illuminated using a solar simulator (AM1.5G, Trisol 1000 W) with a beam diameter of approximately 20 cm. High irradiances were achieved by focusing the simulated solar light with Fresnel lenses (51 mm focal point, 50 mm aperture, Knight Optical, UK) onto a 3.9 mm diameter circular area. This focal spot matched the electroactive area of the photoelectrodes and the receptor (CC-3-UVS, Ocean Optics) of the spectrometer (FLAME-S-XR1, Ocean Optics) used for intensity readings.

To systematically control the irradiance, Fresnel lenses were combined with varying neutral density filters (63%, 40%, and 10% transmittances; metallic ND filter UV grade, Knight Optical). This configuration yielded irradiances of 12, 43, 76, and 120 suns, which were evaluated alongside a baseline of 1 sun (1 kW m^−2^) achieved without concentrating optics.

The PEC cells were controlled by a multichannel potentiostat (EmStat4 HR, PalmSens). Each cell was configured with a reversible hydrogen electrode (RHE, HydroFlex/Gaskatel) as the reference and a platinum wire as the counter electrode. A 0.1 M Na2SO4 (99%, Alpha Aesar) solution, with its pH adjusted to 13.4 by adding NaOH (>99%, Carl Roth), served as the electrolyte. Electrolyte solutions were continuously circulated through the cells *via* a peristaltic pump at a flow rate of 1.6 cm^3^ s^−1^. Operating temperatures were regulated by immersing the electrolyte reservoirs in a temperature-controlled water bath and were continuously monitored with a thermocouple placed directly inside the PEC cell. Stable temperatures at 17 °C, 24 °C, 34 °C, 38 °C, 44 °C and 50 °C were used for the tests with 17 °C and 50 °C representing the lower and upper technical limit. Standard electrochemical testing included seven-hour long chronoamperometry (CA) at a constant applied potential of 1.23 V *vs.* RHE, cyclic voltammetry (CV) conducted at 20 mV s^−1^, and EIS using a 10 mV amplitude over a frequency range of 10^5^ to 0.1 Hz. To ensure reproducibility two identical photoanodes were measured for each parameter variation, where the aberrance was in all cases below 10%.

### 2.8 Performance and stability metrics

To determine photoanode efficiency and stability, the obtained chronoamperometry curves were fitted with the sum of two exponential decay functions (eqn (2)).^36^2
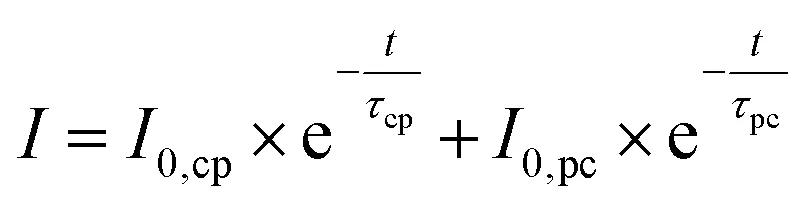


where *I*_0,cp_ represents the predominantly capacitance induced and *I*_0,pc_ represents the photogenerated current at *t* = 0, while *τ*_cp_ and *τ*_pc_ denote the time constants of their respective decays. Hence to compare photoanode efficiency under the different conditions *I*_0,pc_ was used. The stability was assessed using the current retention Ret_CA_, *i.e.* the ratio between the current density at the end of each seven-hour chronoamperometry measurement (*I*_end_) and the photogenerated current at *t* = 0 *I*_0,pc_ (eqn (3)).3
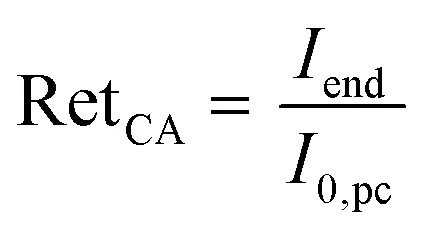


To assess the total energy output of the LTON-based photoanodes, the accumulated charge (*Q*_acc_) after seven-hour chronoamperometry was determined by integration of the measured chronoamperometry curves (eqn (4)).4
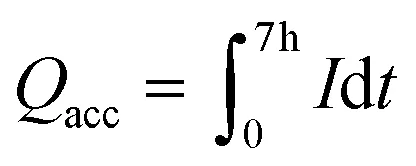


### 2.9 Electrical impedance spectroscopy

For EIS on the LTON-based photoanodes a potentiostat (Autolab, Metrohm) with the software NOVA 1.10 is used. The applied potential is varied from 0.9 V to 1.4 V *vs.* RHE in 0.1 V steps with an AC amplitude of 10 mV and in the frequency range of 0.1–20 kHz. The measurements are performed under illuminated and dark conditions and at electrolyte temperatures of 25 °C, 35 °C and 45 °C. The resulting Nyquist plots are fitted using an equivalent circuit consisting of a serial resistance combined with two RC circuits (Fig. S1) as proposed for PEC electrodes in several studies in the literature.^19,21,40^

## 3 Results and discussion

### 3.1 LTON photoanodes

**Fig. 1:**
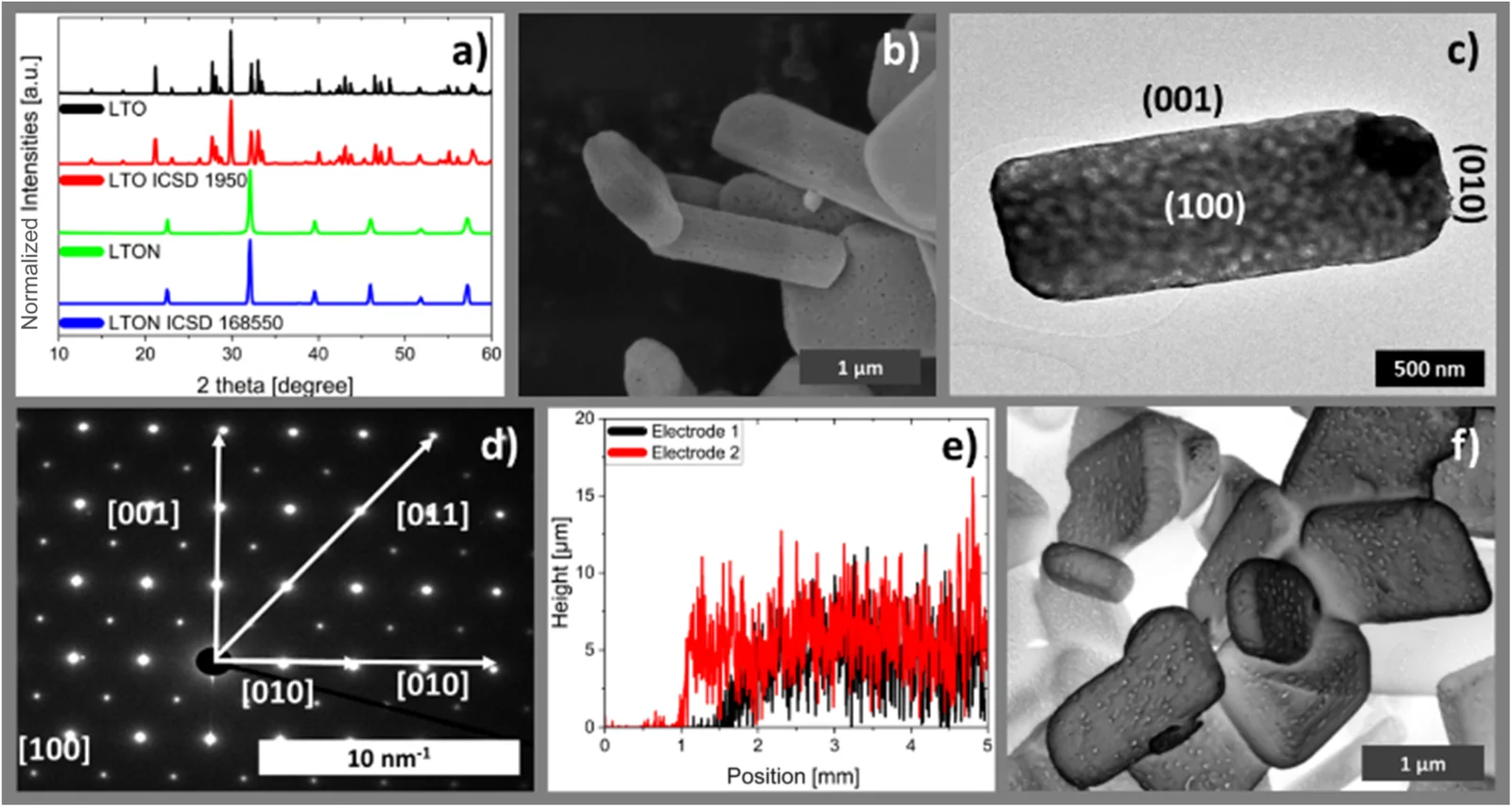
(a) XRD patterns of the synthesized LTO powder and the LTO ICSD 1950 (ref. 1) as well as LTON powder and LTON ICSD 168550,^9^ (b) SEM image of LTON powder (scale bar: 1 µm), (c) BF-TEM (scale bar: 500 nm), (d) SAED pattern of LTON powder (scale bar: 10 nm^−1^), and (e) thickness of the LTON film with respect to the substrate of selected LTON-based photoanodes obtained by profilometry. By integration over the position an average thickness of 6 µm was calculated for the LTON film, and (f) SEM image of a LTON-based photoanode (scale bar: 1 µm).

Phase purity of the synthesized LTO and LTON powders was ensured by performing XRD and by comparing the experimental diffractograms to the corresponding reference diffractograms (Fig. 1a). In agreement with the literature, micrometre-sized brick-shaped porous particles were observed when imaging the powder *via* SEM and TEM (Fig. 1b and c). Due to these pores, the synthesized LTON powders exhibit enhanced surface areas between 10 and 15 m^2^ g^−1^ (Fig. S2). Investigations using selected area (electron) diffraction showed that these brick-shaped particles are monocrystalline with the large facets oriented in the [100] direction while the side facets are oriented in the 〈010〉 and 〈001〉 directions (Fig. 1c and d). After electrophoretic deposition around 6 µm thick, opaque films consisting of irregularly stacked LTON particles are obtained on top of the conductive FTO substrate (Fig. 1e). The conductivity of the LTON film was increased by the formation of TiO_2_ necks between the LTON particles (Fig. 1f).^41,42^

In the HAADF and the elemental EDX maps of Ti and La a porous network was observed inside the LTON particles (Fig. S3a–c). Also, the presence of Ta, Ni, and Co on the LTON particles was evidenced in the corresponding elemental maps (Fig. S3d and e). All three elements are detected in both in the open pores and at the particle surface. Consistent with previous reports the distribution of Ta appeared relatively homogeneous, whereas Ni and Co exhibited a more heterogeneous distribution, associated with nanoparticles.^34,36,43^ According to literature reports the TaCl_5_ treatment leads to the formation of a protective Ta_2_O_5_ layer on top of the photoanode^34^ while the treatment with Ni(NO_3_)_2_ and Co(NO_3_)_2_ results in the formation NiO_*x*_ and CoO_*x*_ nanometre-sized cocatalyst particles on top of the LTON particles.^34^ It has been proposed that the decoration of LTON photoanodes with these nanostructures improved the overall performance of the photoanodes because of increased stability and facilitated charge transfer.^44,45^ Our results show that some Co, Ni and Ta containing species were deposited into the pores of LTON in addition to cocatalyst or protective layer formation at the surface.

To investigate the efficiency and stability of the LTON-based photoanodes under standard conditions seven-hour chronoamperometry measurements were performed under an irradiance of 1 sun and at an electrolyte temperature of 34 °C (Figure S4). The resulting curve progression showed a sharp initial decay within the first minutes, followed by a gradual decline over the remaining seven hours, which is similar to the pattern previously described by Hörndl *et al.*^36^ By using the same analysis method as proposed in ref. 31, *i.e.* by fitting the chronoamperometry curves using eqn (2), an *I*_0,pc_ value of 1.69 ± 0.12 mA cm^−2^ was obtained for the photoanode efficiency, along with a current retention of 50.6 ± 1.7% (eqn (3)) after 7 h as a measure of its stability (Table S1).

### 3.2 Influence of irradiance on photoanode efficiency and stability

**Fig. 2:**
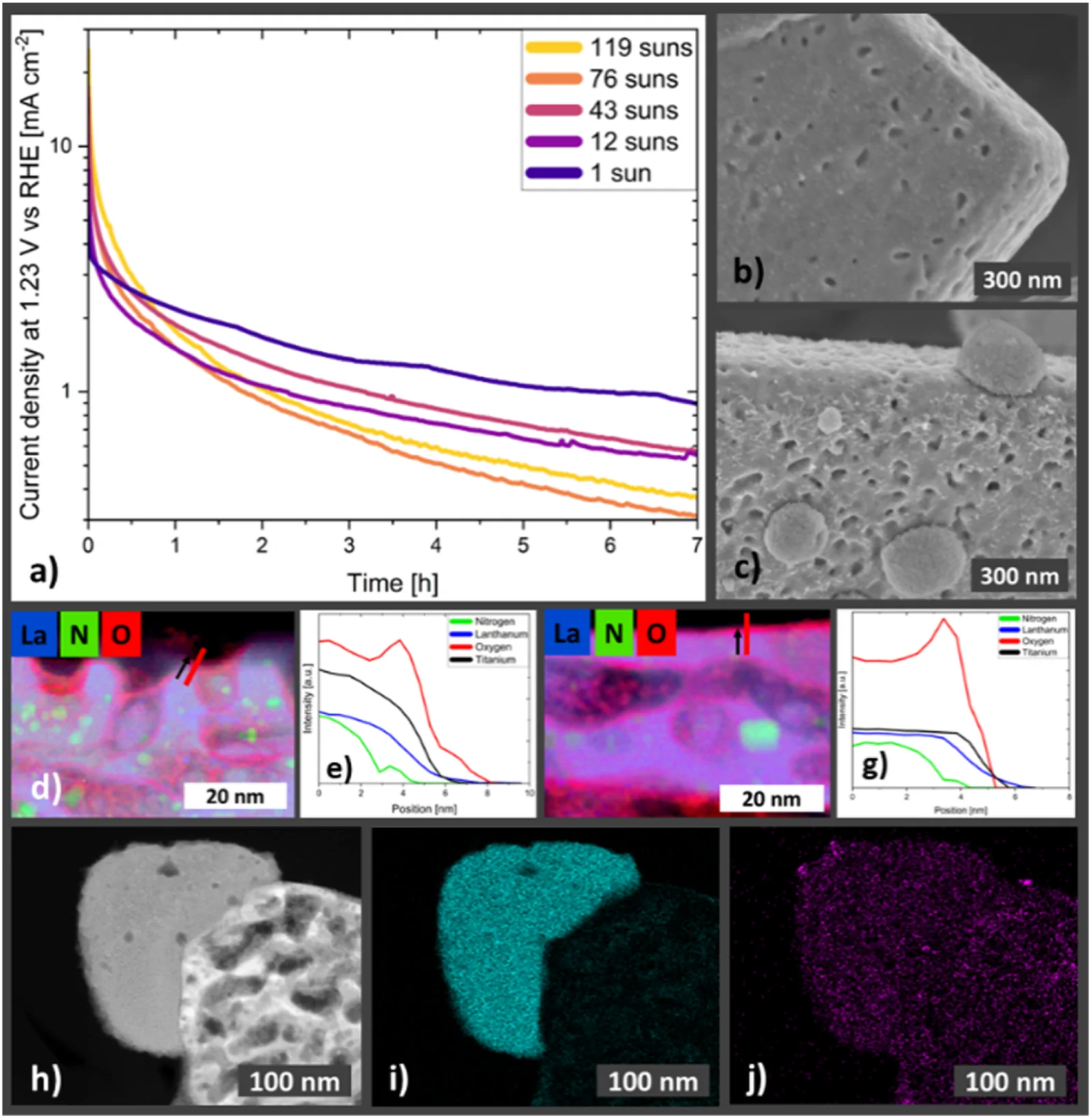
(a) Chronoamperometry curves of LTON-based photoanodes at different irradiances. SEM images of (b) a pristine LTON-based photoanode and (c) after seven-hour chronoamperometry under 119 suns (scale bar: 300 nm). EELS composite maps of the La-M edge, O-K edge and the N-K edge of lamellae extracted from LTON photoanodes after seven-hour chronoamperometry at (d) 1 sun and (f) 119 suns (scale bar: 20 nm). (e) and (g) Line profiles of the integrated La (turquoise), O (blue) Ti (black) and N (green) edge intensities (red lines in the corresponding ELLS map indicate the location of the line profiles). (h) HAADF image of lamellae extracted from a photoanode after seven-hour chronoamperometry under 119 suns. (i) Ta and (j) Ni elemental maps of the same cross sections (scale bar: 100 nm).

To enable a meaningful and systematic investigation, the irradiance was varied over a wide range: 1, 12, 43, 76 and 119 suns. Seven-hour chronoamperometry measurements at 1.23 V *vs.* RHE were used to assess the efficiency and stability of LTON-based photoanodes as a function of the irradiance. During these measurements the electrolyte temperature was maintained at a constant temperature of 32 °C for all irradiances to ensure a direct correlation between potential changes of photoanode efficiency and stability and the irradiance. Independent of the irradiance, all chronoamperometry measurements showed the same characteristic curve progression, that is a sharp decay in the first minutes followed by a more gradual decay for the remaining measurement, that was already described by Hörndl *et al.*^36^ (Fig. 2a). For further analysis the chronoamperometry curves were fitted using eqn (2) and the photogenerated currents at *t* = 0 (*I*_0,pc_) and the current retentions Ret_CA_ were used as metrics for the efficiency and stability of the LTON-based photoanodes. These values are listed in Table 1, while in Table S2 a summary of all fitting parameters is provided. First, we focus on the efficiency, *i.e. I*_0,pc_. The LTON-based photoanodes showed an increase of the photogenerated current with increasing irradiance, similar to previous studies on GaInP_2_,^24^ Fe_2_O_3_ (ref. 25) and LaFeO_3_.^27^ This was expected, since higher irradiances generally lead to higher charge generation rates.

**Table 1:** Calculated photocurrent densities at *t* = 0, photocurrent densities at the end of a 7h experiment, current retentions and accumulated charge of LTON-based photoanodes obtained from chronoamperometry under different irradiances

Irradiance [suns]	*I*_0, pc_ [mA cm^−2^]	*I*_end_ [mA cm^−2^]	Ret_CA_ [%]	*Q*_acc_ [mAh cm^−2^]
1	1.82	0.90	49.5	10.51
12	1.68	0.55	32.7	7.10
43	2.49	0.57	22.9	8.81
76	2.54	0.31	12.2	6.54
119	3.21	0.37	11.5	8.13

However, the 1.8-fold increase in *I*_0,pc_ observed for 119 suns, compared to 1 sun, was surprisingly small. This might be explained by a non-linear increase of recombination and transport losses with irradiation intensity. Interestingly, the capacitive current at *t* = 0 (*I*_0,cp_) exhibited a more than seven-fold increase (Table S2) for the same irradiance increase. This suggests that most of the observed increase in the initial current at high irradiances (Fig. 2a) was driven by a short-lived capacitive current, rather than by the photogenerated current. This hypothesis is further supported by the observation that, after just one hour, the photoanode measured at 1 sun already exhibited the highest current density, surpassing all other photoanodes.

With respect to stability, a sharp decline in current retention Ret_CA_, from 49.5% at 1 sun to 11.5% at 119 suns, was observed, indicating a strongly reduced photoanode stability at higher irradiances. A similar observation was previously reported in a qualitative study on the stability of BiVO_4_-based photoanodes.^22,39^

To investigate the origin of this sharp decay in Ret_CA_ with increasing irradiance, the LTON-based photoanodes were investigated after seven-hour chronoamperometry at different irradiances using XRD, SEM, STEM-EDX/EELS and HREM. Thus, we first discuss the observed changes, or lack thereof, in the bulk LTON, followed by an assessment of the surface-related characterisation. The XRD patterns of LTON-based photoanodes after seven-hour chronoamperometry obtained under 1 sun and 119 suns did not show any changes with respect to pristine LTON photoanodes (Fig. S5), suggesting that neither the crystal structure nor the phase composition of bulk LTON was affected by illumination, even at irradiances as high as 119 suns. Regarding morphology, hardly any changes were detected by SEM compared to the pristine samples after seven-hour chronoamperometry under an irradiance of 1 sun (Figure S6a, b). Meanwhile, the formation of nanoparticles (with sizes ranging from 20 to 70 nm) was observed on top of the LTON particles after seven-hour chronoamperometry under an irradiance of 12 suns (Fig. S6c). Further SEM studies showed that with increasing irradiance during chronoamperometry these spherical particles increased further in both size and quantity (Fig. 2b, c, S6 and S7). Aside from the formation of these spherical particles, the LTON particles themselves appeared morphologically unchanged independent of the irradiance. For an in-depth investigation, TEM lamellae were extracted from the photoanodes after seven-hour chronoamperometry under 1 and 119 suns. Focusing first on LTON, neither the HAADF images nor the corresponding Ti and La elemental maps indicated morphological or compositional changes (Fig. S8). Although these results are in agreement with literature reports showing that bulk LTON remained stable under standard operating conditions, it is somewhat surprising that this stability was preserved even at elevated irradiance.^36,46^

To investigate the particle surfaces more closely, HREM images were acquired. Comparing the crystallinity of LTON particles of pristine photoanodes with particles of photoanodes after seven-hour chronoamperometry under irradiances of 1 sun and 119 suns (Figure S9), modifications of the crystal structure were observed close to the particle surface (2 to 5 nm), indicating the formation of a different phase. The chemical composition of this surface layer was investigated using EELS. In the EELS maps, increased O K-edge intensities were observed in the outermost 2–5 nm for LTON particles from photoanodes after seven-hour chronoamperometry at irradiances of 1 sun and 119 suns (Fig. 2d, f and S10). This effect is visible more quantitatively in the line profiles acquired across the respective particle surfaces. As displayed in Fig. 2e and g the O K-edge intensity increase is accompanied by a decrease in N K-edge intensity in the outermost 2–5 nm. These observations are coherent with the formation of oxide layers at the particle surfaces, a degradation process that was reported before for LTON photoanodes under standard operating conditions.^36^ Interestingly, neither HREM nor EELS revealed any noticeable differences regarding the thickness of the oxygen rich layer, although the irradiance was varied in a large range, *i.e.* between 1 sun and 119 suns. These results indicate that this degradation process is not strongly influenced by the irradiance within the time scale of this study.

Focusing now on the spherical nanoparticles formed at the surface of LTON particles after illumination, we selected the photoanode exposed to an irradiance of 119 suns for further studies, because it showed the largest effect. Chemical analysis by STEM-EDX elemental maps indicated that these nanoparticles consisted mainly of TaO_*x*_ (Fig. 2h and i). Comparing the Ta elemental contrast in the pores of the LTON particles with that in the nanoparticles suggests that most of the Ta present on the LTON photoanode is now located within these spherical particles. Therefore, we propose that the formation of these spherical nanoparticles is caused by the dissolution of Ta-species, followed by their redeposition on the particle surface forming spheres. The increase in the size and number of these nanoparticles with irradiance is an indication of the accelerated dissolution and redeposition of Ta with increasing irradiance. Meanwhile, Ni and Co were found to be homogeneously distributed over both the spherical particles and the pores of the LTON particles (Fig. 2j and S11). Therefore, we assume that some of the Ni and Co have been moved from the LTON particles to the spherical particles by similar dissolution/redeposition mechanisms. A similar effect, but much less pronounced with respect to the observed morphological changes, *i.e.* the nanoparticle formation, was reported by Hörndl *et al.* for LTON photoanodes under standard operating conditions. In this article the authors proposed that redeposited Ni and Co species were less effective for water oxidation as they were located on already oxidized LTON surfaces after redeposition.^36^ They claimed that this effect was at least partly responsible for the observed decrease in the current density with time during chronoamperometry. In our study we found a higher number and larger spherical particles containing Ta, Ni and Co suggesting an acceleration of the dissolution/redeposition mechanisms with increased irradiance. We assume that this leaves fewer Ni and Co species on the still unoxidized LTON surface. Hence, it is very likely that the observed stability decrease of the LTON-based photoanodes with increasing irradiance (Table 1) was caused by a significant acceleration of the cocatalyst and protection layer dissolution/redeposition mechanism. These findings confirm the correlation between cocatalyst dissolution/redeposition and photoanode deactivation that was suggested previously.^36^

To further investigate the influence of the irradiances on these mechanisms, the concentrations of La, Ti, Ta, Ni and Co in the electrolyte were determined by ICP-MS after seven-hour chronoamperometry under different irradiances (1 sun – 119 suns) (Table 2 and Fig. S12). The concentration of La was below the detection limit of 0.1 µg L^−1^ for all investigated samples. In combination with the results of the SEM and STEM-EDX investigations this observation confirmed that the stability of bulk LTON was not affected by the increase of the irradiance. Hence, the Ti concentrations were assigned to the dissolution of the TiO_2_ necking, while the Ta and Co concentrations were attributed to the dissolution of the Ta_2_O_5_ layer and the CoO_*x*_ cocatalyst nanoparticles. For an increase in the irradiance from 1 sun to 119 suns, an increase in the final concentrations by factors of 3 (Ti), 8 (Ta) and 2.5 (Co) was observed (Table 2). In combination with the observations made by SEM and STEM-EDX, we conclude that the decreased stability at higher irradiances stems from increased loss of cocatalysts available for charge extraction and separation due to an acceleration of their dissolution and redeposition with the irradiance.^36^

**Table 2:** Concentration changes of La, Ti, Ta, Ni and Co in the electrolyte after seven-hour chronoamperometry under different irradiances

Irradiance [suns]	La [µg l^−1^]	Ti [µg l^−1^]	Ta [µg l^−1^]	Co [µg l^−1^]
1	<0.1	2.0	1.6	0.5
12	<0.1	6.3	14.9	1.5
43	<0.1	3.4	2.5	1.1
76	<0.1	6.6	6.9	0.9
119	<0.1	6.4	12.4	1.3

In summary, the increase of the irradiance from 1 sun to 119 suns during seven-hour chronoamperometry led to a (surprisingly small) increase in I_0,pc_ of the LTON-based photoanodes by 80%. However, this increased initial current density at 119 suns was quickly lost because of a substantial decrease in the current retention Ret_CA_ by almost 40% in comparison to the retention at an irradiance of 1 sun. We found evidence that the lower photoanode stability under these conditions was due to accelerated dissolution/redeposition processes of the cocatalysts and protection layer. We have further demonstrated that this effect is the main reason for the decreased stability of the LTON-based photoanodes at higher irradiances, while the surface oxidation of LTON is not affected in a significant manner by increased irradiances.

### 3.3 Influence of the electrolyte temperature on photoanode efficiency and stability

**Fig. 3:**
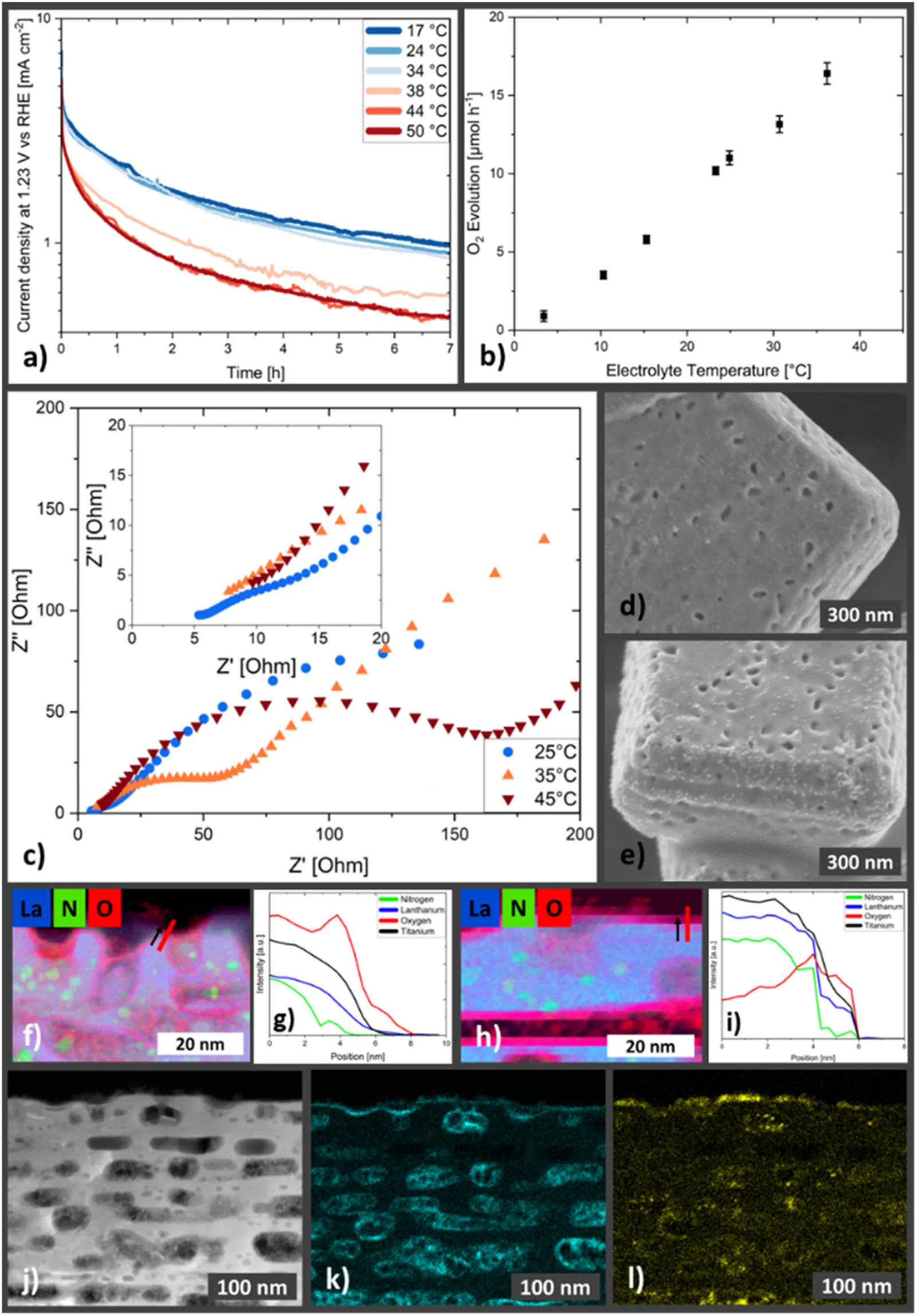
(a) Chronoamperometry curves of LTON-based photoanodes at different electrolyte temperatures. (b) Evolved amount of oxygen within one hour by 20 mg LTON under 1.5 AMG illumination. (c) EIS measurements of LTON-based photoanodes at different electrolyte temperatures and under AMG 1.5 illumination at a potential of 1.2 V *vs.* RHE. SEM images of (d) a pristine LTON-based photoanode and (e) after seven-hour chronoamperometry at an electrolyte temperature of 44 °C (scale bar: 300 nm). EELS composite maps of the La-M edge, O-K edge and N-K edge of lamellae extracted from the LTON photoanode after seven-hour chronoamperometry at electrolyte temperatures of (f) 34 °C and (h) 44 °C (scale bar: 20 nm). (g) and (i) Line profiles of the integrated La (turquoise), O (blue) Ti (black) and N (green) edge intensities (red lines in the corresponding ELLS map indicate the location of the line profiles). (j) HAADF image of lamellae extracted from a LTON based photoanode after seven-hour chronoamperometry at an electrolyte temperature of 44 °C. (k) Ta and (l) Ni elemental maps of the same cross sections (scale bar: 100 nm).

To investigate the influence of temperature on the efficiency and stability of the LTON-based photoanodes seven-hour chronoamperometry measurements at 1.23 V were performed under 1 sun illumination at different electrolyte temperatures ranging from 17 °C to 50 °C (Fig. 3a). Independent of the electrolyte temperature all chronoamperometry measurements showed similar curve progressions: a sharp decay in the first minutes, followed by a more gradual decay during the remaining seven hours. This agrees with the observations reported in a previous study.^31^ Similarly, eqn (2) was then used to fit the chronoamperometry curves and estimate I_0,pc_ and Ret_CA_, the values of which are listed in Table 3, while Table S3 contains all fitting parameters.

**Table 3:** Maximum photocurrent densities, current retentions and accumulated charge of LTON-based photoanodes obtained from seven-hour chronoamperometry at different electrolyte temperatures

Electrolyte temperature [°C]	*I*_0, pc_ [mA cm^−2^]	*I*_end_ [mA cm^−2^]	Ret_CA_ [%]	*Q*_acc_ [mAh cm^−2^]
17 °C	1.97	0.98	49.8	11.09
24 °C	1.87	0.90	48.1	10.48
34 °C	1.71	0.86	50.3	10.25
38 °C	1.25	0.58	46.4	6.70
44 °C	1.03	0.47	45.6	5.67
50 °C	1.12	0.47	42.0	5.62

Focusing first on photoanode efficiency, we observed that the *I*_0,pc_ of 1.97 mA cm^−2^ decreased by almost 50% to 1.12 mA cm^−2^, as the electrolyte temperature increased from 17 °C to 50 °C. This result is surprising, since an increase in reaction rates is generally expected with increasing temperature according to the Arrhenius law. Although literature reports on the temperature dependence of photoanode efficiency are not consistent, often varying with the photoelectrode architecture and applied potential, most investigated materials (*e.g.,* CuWO_3_ or BiVO_4_) exhibit increased efficiency at higher electrolyte temperatures, aligning with kinetic considerations.^19,20,22,39^ However, Valenza *et al.* found the same trend for Fe_2_O_3_-based photoanodes, as we have observed for our LTON based photoanodes.^21^ The authors attributed this effect to an increase in surface charge recombination resulting in lower conversion efficiencies. Since the observed temperature related behaviour of LTON-based photoanodes is different compared to most of the literature, it is worthwhile to further explore its origin.

Since photoelectrochemical efficiency is governed by a complex balance of several effects such as optical absorption, charge separation, charge recombination, charge transport to the back contact and to the surface and catalytic reaction rate, the temperature dependences of all of them need to be considered. This complexity can be simplified by categorizing these phenomena into particle photoactivity related aspects and electrode, *i.e.* transport, related effects. Since we work with particle-based electrodes, it is possible to study the photoactivity of LTON particles without electrode related effects by investigating the photocatalytic activity of the LTON particles as a function of temperature. For this purpose the oxygen evolution rates of LTON particle suspensions were measured in the presence of a sacrificial agent at electrolyte temperatures ranging from 3 °C to 36 °C, where the lowest oxygen evolution rate was obtained at 3 °C with 0.9 ± 0.35 µmol h^−1^ and the highest was obtained at 36 °C with 16.4 ± 0.7 µmol h^−1^, resulting in a more than tenfold increase in the oxygen evolution rate over the investigated temperature range (Fig. 3b and Table S4). A similar correlation between the electrolyte temperature and the photocatalytic activity of the photocatalyst was also observed by Chen *et al.* for TiO_2_ nanoparticles.^47^ On plotting the measured oxygen evolution rates in an Arrhenius plot (Fig. S13) we found a slight deviation from the expected linear behaviour. This is an indication that the exponential increase of the reaction rate as expected according to the Arrhenius law is modulated by effects such as charge carrier recombination. Charge carrier recombination was discussed in the literature as a potential reason for photoactivity decrease with increasing temperature, reducing the expected exponential increase to a nearly linear one in our case.^21^ Nevertheless, an increase in the electrolyte temperature led to increased photoactivity on the LTON particle level. This is an indication that the observed decreasing efficiency of the LTON-based photoanodes with increasing electrolyte temperature is likely to be caused by a dominating electrode related effect. In addition to charge generation the performance of a photoanode is also strongly influenced by its charge transportation and separation efficiency.^48^ While an increase in temperature is known to enhance both charge carrier recombination and surface reaction rates, the net effect on performance depends on which of these two competing processes predominates in the specific material system.^48,49^

To investigate the mechanism behind this efficiency disparity, EIS was performed at 25 °C, 35 °C and 45 °C electrolyte temperatures. The resulting Nyquist plots are composed of two compressed semicircles (Fig. 3c, S14 and S15). Similar Nyquist plot shapes have already been reported for other photoanode types under similar conditions.^19,21,40^ First, we analysed the onset of the Nyquist plots and observed a shift to higher impedance values with increasing electrolyte temperature, independent of the applied potential (Table S5). The high frequency impedance is commonly associated with ohmic resistances that are in series in the whole system (*i.e.* the electrolyte, the conductive substrate, electrical contacts and the composite photoelectrode system itself.^19,21,50^ Thus, its shift to higher values with increasing electrolyte temperature indicated an increased resistance of either the electrolyte, the conductive substrate or the semiconductor at elevated temperatures. Focusing next on the high frequency semicircles, an increase in their diameters with increasing electrolyte temperature was observed independent of the applied potential (Fig. S14 and S15 and Table S6). This semicircle is commonly associated with fast electronic processes occurring within the bulk of the photoanode.^40,50^ Hence, we interpreted the increase in the first semicircle diameters with the electrolyte temperature as an increase in the charge transport resistance in the bulk of the LTON-based photoanodes. In some cases, bulk charge carrier transport resistances are associated with the trapping and detrapping of charges on defects.^43^ Since both the LTON and the TiO_2_ necking are known to have many defects,^41,51^ the measured bulk resistance of the LTON photoanodes is expected to be impacted by the temporary interaction of the charge carriers with the trap states caused by these defects.^40^ Thus, we assume that the observed increase in the charge carrier transport resistance of the LTON-based photoanodes at higher electrolyte temperatures is caused by an increase in the number of accessible defects for trapping/detrapping at higher temperatures. In summary, we propose that the decrease in efficiency of LTON-based photoanodes at elevated temperatures is caused by the increase in the serial resistance of the whole system and the electron trapping/detrapping resistance within the LTON photoanodes with the temperature.

Next, we investigate the stability of LTON photoanodes as a function of temperature using current retention Ret_CA_ as an indicator. With values of 49.8% at 17 °C and 42.0 at 50 °C, the Ret_CA_ of the LTON-based photoanodes decreased with increasing electrolyte temperature. A similar effect of the electrolyte temperature on photoanode stability was also observed by Dias *et al.* on Si-doped Fe_2_O_3_ photoanodes, caused by an accelerated corrosion of the photoanode surface at temperatures above 50 °C.^19^

To investigate the origin of this decline of Ret_CA_ with increasing electrolyte temperature, the LTON-based photoanodes were investigated before and after chronoamperometry using the same methods as applied in the study under different irradiances. Comparing the XRD patterns of LTON-based photoanodes after seven-hour chronoamperometry at electrolyte temperatures of 34 °C and 44 °C did not reveal any changes with respect to the pristine LTON photoanode (Fig. S16). This indicates that the crystal structure of bulk LTON remained intact independent of the used electrolyte temperature. For electrolyte temperatures below 35 °C, no morphological differences were observed by SEM between the pristine photoanodes and those after seven-hour chronoamperometry (Fig. S17a and b). However, at higher electrolyte temperatures, the formation of nanoparticles with an average size of 12 ± 4 nm (measured over 50 particles) was observed on top of the LTON particles (Fig. 3d and e). For further analysis TEM lamellae were extracted from pristine photoanodes and photoanodes after seven-hour chronoamperometry at electrolyte temperatures of 34 °C and 44 °C. The comparison of the different HAADF images and the corresponding La and Ti elemental maps (Fig. S18) revealed no morphological differences with respect to *pristine* LTON regarding porosity and chemical composition. Consistent with the literature these observations confirmed that the bulk of the LTON particles was stable under ambient temperature operating conditions^36,46,52^ and remained stable even at elevated electrolyte temperatures up to 44 °C as shown by our results. Since LTON is known for surface modifications under operating conditions,^36^ HREM was used to investigate the crystallinity of the LTON particles close to the surface in pristine LTON-based photoanodes and after seven-hour chronoamperometry at electrolyte temperatures of 34 °C and 44 °C (Fig. S19). As before, a change in the crystal structure was observed close to the surface (2–5nm) of the two photoanodes after seven-hour chronoamperometry indicating the formation of a different phase near the particle surface. EELS measurements confirmed the existence of an oxygen rich layer in the outermost 2–5 nm of the LTON particle samples, as shown in the increased O K-edge intensities in the EELS maps (Fig. 3f, h and S20) and in the line profiles (Fig. 3g and i). In addition, neither HREM nor EELS revealed any significant differences regarding the thickness of the oxygen rich layers, within the precision of our measurements, when comparing photoanodes after operation at 34 °C and at 44 °C. Hence, we conclude that the degradation process related to surface oxidation is a self-limiting process, which is not significantly affected by the electrolyte temperature.

As already discussed above, another surface related degradation process in LTON-based photoanodes is linked to the cocatalysts. On investigating the cocatalyst distribution by STEM-EDX, the corresponding Co elemental maps showed an increased intensity at the LTON particle surface after seven-hour chronoamperometry at 44 °C in comparison to the pristine photoanode (Fig. 3j and k). In addition, Ni and Ta were detected at the particle surface, but their respective distributions over the whole particle, including the pores, showed no obvious change with the electrolyte temperature (Fig. 3l and S21). In combination with the SEM images where the formation of small nanoparticles at the particle surface was observed, these findings indicated that these nanoparticles consisted of Co, Ni, and Ta species and were formed through their dissolution and redeposition. The increased size of these particles at higher electrolyte temperatures suggested that an increase of the electrolyte temperatures also accelerated the dissolution/redeposition rates of the cocatalysts and the protective layer, in a similar manner as previously observed in irradiance-dependent studies.^36^

To further investigate the influence of the electrolyte temperature on this cocatalyst related degradation process the concentrations of La, Ti, Ta, Ni and Co in the electrolyte were determined by ICP-MS after seven-hour chronoamperometry at electrolyte temperatures between 17 °C and 50 °C (Table 4 and Figure S22). We observed in all cases significant concentrations of Ti, Ta and Co in the electrolyte. In agreement with the STEM-EDX/EELS investigations the absence of La in the electrolyte suggested that bulk LTON was stable independent of the electrolyte temperature. In the absence of La, we attributed the observed Ti in the electrolyte to dissolution of the TiO_2_ necking during chronoamperometry. Furthermore, we assumed that the presence of Ta and Co in the electrolyte was caused by the dissolution of the Ta_2_O_5_ layers and the CoO_*x*_ cocatalyst nanoparticles. It is noteworthy that the final concentrations of Ti, Ta and Co increased by factors of 65 (Ti), 7 (Ta) and 5 (Co) for an increase of the electrolyte temperature from 16 to 50 °C, while factors of 3 (Ti), 8 (Ta) and 2.5 (Co) were obtained when comparing 1 sun to 119 suns. Thus, the concentrations of Ti and Co after seven-hour chronoamperometry increased significantly stronger as a function of temperature than of the irradiance. Interestingly the opposite trend was observed for photoanode stability, *i.e.* a decrease in the Ret_CA_ by only 8% for an increase of the electrolyte temperature from 17 °C to 50 °C and a decrease by almost 40% for an increase of the irradiance from 1 sun to 119 suns.

**Table 4:** Concentrations of La, Ti, Ta and Co in the electrolyte after seven-hour chronoamperometry at different electrolyte temperatures

Electrolyte temperature [°C]	La [µg l^−1^]	Ti [µg l^−1^]	Ta [µg l^−1^]	Co [µg l^−1^]
16	< 0.1	0.4	0.3	0.4
25	< 0.1	2.9	0.8	0.3
34	< 0.1	2.0	1.6	0.6
38	< 0.1	4.7	1.6	0.8
44	< 0.1	10.1	2.3	1.1
50	< 0.1	27.9	n.a.	1.9

**Fig. 4:**
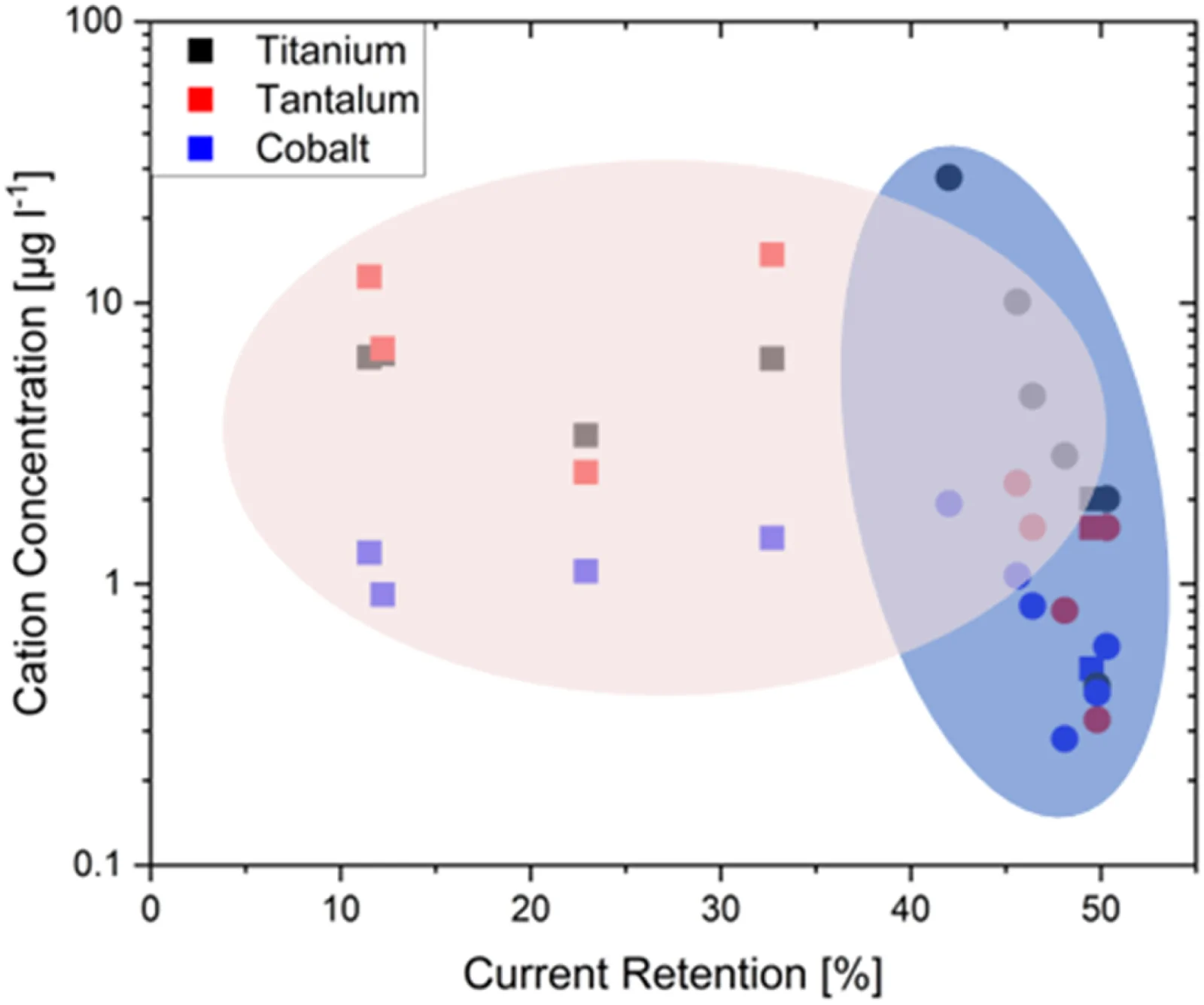
Final cation concentration of Ti, Ta and Co after seven-hour chronoamperometry as a function of the corresponding current retention. Squares/circles indicate concentrations after chronoamperometry at different irradiances/electrolyte temperatures, while pink/blue areas are respective guides to the eye.

This discrepancy shows that the respective Ti, Ta, and Co concentrations in the electrolyte after seven-hour chronoamperometry are not good indicators of the overall stability of the LTON-based photoanodes. This absence of a correlation was visualized in a plot of the final cation concentration as a function of the Ret_CA_ (Fig. 4). Considering that the cocatalyst shapes changed during chronoamperometry, these results confirmed that apart from dissolution, redeposition is an important effect. Since Ti, Ta and Co were not only dissolved but also redeposited, their respective concentrations in the electrolyte represented simply the difference between the dissolved and the redeposited amounts. Therefore, we propose that the measured concentrations after the seven-hour chronoamperometry were not indicative of the total dissolution of the different elements, but rather represented equilibrium concentrations between the dissolution and redeposition reactions of Ti, Ta and Co. Hence if both dissolution rates and redeposition rates increased simultaneously, this should result in larger amounts of cocatalysts being dissolved during chronoamperometry without necessarily causing changes in the cation concentrations after seven-hour chronoamperometry. Our previous observations suggested that an increase in the irradiance led to a stronger acceleration of the cocatalyst dissolution than an increase in the electrolyte temperature. However, as this increased dissolution rate was not reflected by the final concentrations of Ti, Ta and Co in the electrolyte after seven-hour chronoamperometry, we propose that the irradiance also led to an accelerated redeposition of the cocatalysts and the protective layer. The consequences of these accelerated dissolution/redeposition mechanisms show in the morphological and compositional changes of the cocatalyst nanoparticles and the protective layer (Fig. 2 and S6, S7). In summary, while the photocatalytic activity of the LTON particles increased with increasing temperature, this effect was counteracted at the electrode level by a decline in the charge carrier transport properties of the photoelectrode. As a result, the overall efficiency of the photoanode decreased with increasing electrolyte temperature. Furthermore, an accelerated loss of the cocatalysts and the Ta_2_O_5_ protective layer with increasing temperature resulted in a decreased stability of the LTON-based photoanodes at elevated electrolyte temperatures. Consequently, the most favourable operating point for seven-hour chronoamperometry was obtained at an electrolyte temperature of 17 °C under 1 sun irradiation.

**Fig. 5:**
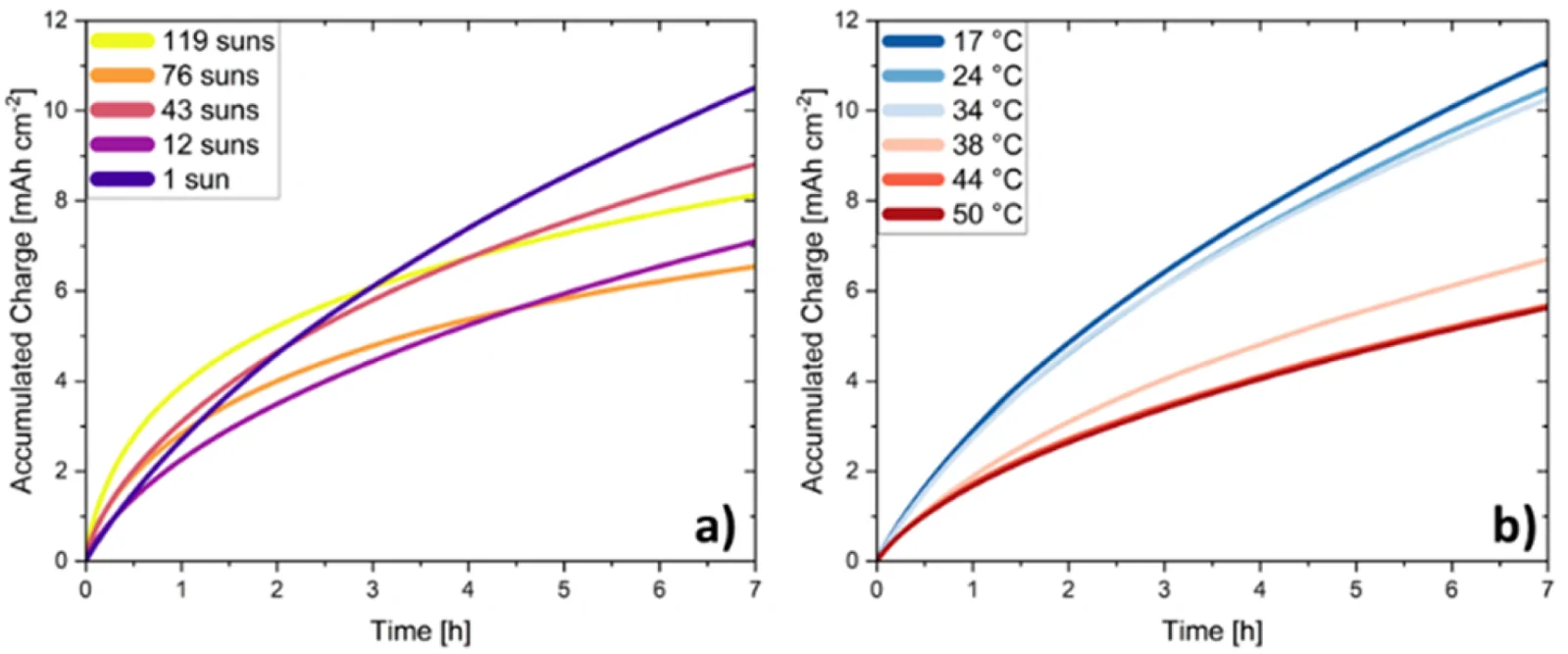
Accumulated charge after seven-hour chronoamperometry (a) under different irradiances and (b) at different electrolyte temperatures (eqn (4)).

Since the aim of this study was to identify the most favourable operating point for large energy output, we calculated the generated charge as a function of time by integrating the current densities obtained during seven-hour chronoamperometry. We used the total charge obtained after seven hours as a measure for energy output (Fig. 5), since it is correlated with the hydrogen generation under the assumption that the photoanodes were coupled to an ideal photocathode at 100% faradaic efficiency. Focusing first on different irradiances, we found that the faster degradation at higher irradiances quickly offset the initially higher photocurrents. Therefore, the highest accumulated charge after seven-hour chronoamperometry was obtained with an irradiance of 1 sun, exceeding the accumulated charge at 119 suns by approximately 20%. Given the almost three times higher final current after the chronoamperometry under 1 sun (Table 1) this performance gap is expected to widen further for longer measurement durations indicating that for maximizing the accumulated charge of the LTON-based photoanodes an irradiance of 1 sun should be used. For higher electrolyte temperatures we found that the increase of the electrolyte temperature to 50 °C resulted in a reduction of the accumulated charge by almost 50%. Consequently, the total energy output at 17 °C was twice as high as that at 50 °C. Given the fact that after 7 h the sample operated at 17 °C exhibited the highest final current together with the sample operated at 34 °C the highest current retention (Table 3), it is reasonable to assume that also for longer measurement durations an electrolyte temperature of 17 °C should yield the highest accumulated charge. Hence, due to the decreasing efficiency and stability of LTON-based photoanodes with increasing electrolyte temperature, they should be operated at low electrolyte temperatures to maximize their energy conversion efficiency. Considering the screened parameter range, the most promising set of operating parameters are 1 sun irradiance and 17 °C electrolyte temperature.

## 4 Conclusions

The influence of irradiance and electrolyte temperature on the efficiency and stability of LTON-based photoanodes was investigated by performing chronoamperometry with irradiances ranging from 1 sun to 119 suns and electrolyte temperatures ranging from 17 °C to 50 °C. The irradiance increases from 1 sun to 119 suns resulted in an initial increase in the efficiency, *i.e.* the photogenerated current at *t* = 0 *I*_0,pc_ increased by 76% and was accompanied by a decrease in the stability Ret_CA_ by almost 40%. The strong increase in the initial current density observed in the experimental chronoamperometry curves was mainly driven by an increase in the short-lived capacitive current and thus not very sustainable. Investigations of the photoanodes after seven-hour chronoamperometry using XRD, SEM, STEM-EDX/EELS, HREM and ICP-MS revealed that bulk LTON remained stable even at high irradiances, while the surface was passivated by a 2–5 nm thick oxide layer at all irradiances. However, the increased degradation at high irradiances was predominantly caused by a sharp acceleration of the cocatalyst and Ta_2_O_5_ layer dissolution/redeposition mechanisms resulting in substantial morphological changes in the cocatalyst nanostructures.

Increased electrolyte temperature resulted in a decrease in efficiency by 43% and in current retention by 8%. While oxygen evolution rate measurements on LTON particles indicated an increase in the photocatalytic activity with electrolyte temperature, EIS confirmed that the decrease in efficiency at higher electrolyte temperatures was caused by transport related effects at the photoanode level, such as an increase in the serial resistance and the electron trapping/detrapping resistance. Investigations of the photoanodes after seven-hour chronoamperometry using XRD, SEM, STEM-EDX/EELS, HREM and ICP-MS confirmed that bulk LTON remained stable at high electrolyte temperatures but was again passivated by 2–5 nm thick surface oxide layers. These experiments also revealed that the decrease in stability was primarily caused by an asymmetric acceleration of the cocatalyst and protective layer dissolution/redeposition mechanisms with the electrolyte temperature favouring the dissolution. Combining the results for higher irradiances and electrolyte temperatures, the LTON-based photoanodes should be operated at low electrolyte temperatures and under non-concentrated illuminations, to maximize their total energy conversion. The most suitable operating point identified in this study was at 1 sun irradiance with an electrolyte temperature of 17 °C at the margin of the technically feasible screening window of our setup. Adapting the photoelectrochemical setup to achieve even lower electrolyte temperatures and lower irradiances has the potential to further enhance both the efficiency and stability of the photoanode.

The findings of this study further highlight the promising stability of LTON, as the observed surface oxidation seemed to prevent complete corrosion even at elevated electrolyte temperatures or irradiances at the time scales of this study. However, the results also reveal a decline in the stability of the cocatalyst and protective Ta_2_O_5_ layer under these same conditions, due to an acceleration of the dissolution redeposition mechanism. To address this, future mitigation strategies for LTON-based photoanodes should prioritise enhancing the resistance of the cocatalyst and protection layer towards dissolution.^36^ One promising approach in this regard is to modify the cocatalyst deposition process to obtain an alternative morphology that might have a higher stability towards dissolution.^53,54^ Another possibility targeting the surface oxidation could be the implementation of an annealing step in a reducing atmosphere after cocatalyst application,^55–57^ as the introduction of oxygen vacancies near the particle surface might impede or at least slow down the observed surface oxidation.^57,58^

## Author contributions

J. H. performed conceptualization, methodology, investigation, formal analysis, writing – original draft. J. Z. performed investigation, writing – review and editing. F. B. performed investigation, writing – review and editing. S. H. performed funding acquisition, writing – review and editing. S. P. performed funding acquisition, supervision, conceptualization, writing – review and editing.

## Conflicts of interest

There are no conflicts to declare.

## Supplementary Material

EL-002-D6EL00054A-s001

EL-002-D6EL00054A-s002

## Data Availability

The data supporting this article have been included as part of the supplementary information (SI). Supplementary information: material and photoanode characterization, photoelectrochemical characterization, fitting results of chronoamperometry and degradation studies. See DOI: https://doi.org/10.1039/d6el00054a.
