# Supplementary material for: Performance and stability of LaTiO2N based photoanodes at varying electrolyte temperatures and irradiances
Source: EES Solar. 2026 Jun 24. Online ahead of print. doi: 10.1039/d6el00054a (PMC13348188; doi:10.1039/d6el00054a)
Supplement: EL-OLF-D6EL00054A-s002 [file EL-OLF-D6EL00054A-s002.pdf]

Supplementary Information (SI) for EES Solar.
This journal is © The Royal Society of Chemistry 2026

Supporting Information

Performance and stability of $LaTiO_2N$-based photoanodes under varying electrolyte temperatures and irradiances

Julian Hörndl[a], Jakub Zalesak[a], Franky E. Bedoya-Lora[b], Sophia Haussener[b], Simone Pokrant[a*]

[a]*Department of Chemistry and Physics of Materials, Paris Lodron University Salzburg, Jakob-Haringer-Str. 2A, 5020 Salzburg, Austria. *E-mail: simone.pokrant@plus.ac.at*

[b]*Laboratory of Renewable Energy Science and Engineering, Ecole Polytechnique Federale de Lausanne, 1015 Lausanne, Switzerland*

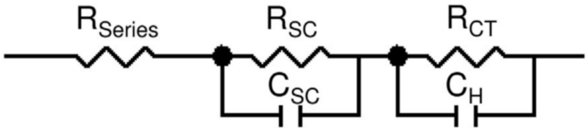

Figure S1: Equivalent electrical circuit used to fit the impedance data. R_{Series} represents the serial resistance of the system, R_{SC} the semiconductor charge resistance, C_{SC} the capacitance of the space charge layer in the semiconductor, R_{CT} the charge transfer resistance at the semiconductor electrolyte interface and C_H the capacitance of the Helmholtz layer in the electrolyte side. [1, 2]

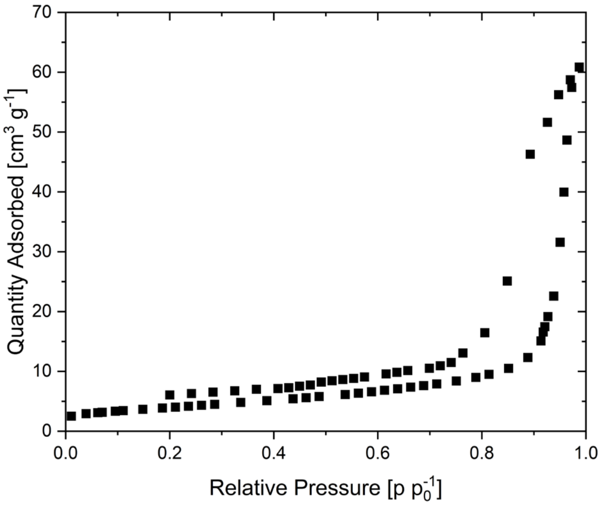

Figure S2: N_2 -Sorption measurement of LTON Powder, carried out with a Micromeritics ASAP 2040 Surface Area and Porosity Analyser. For the measurements liquid nitrogen with a temperature of 77 K was used. Before the measurements the powder was degassed for 2 h at 150 °C. The obtained BET Surface area is 14.2 g m^{-2}.

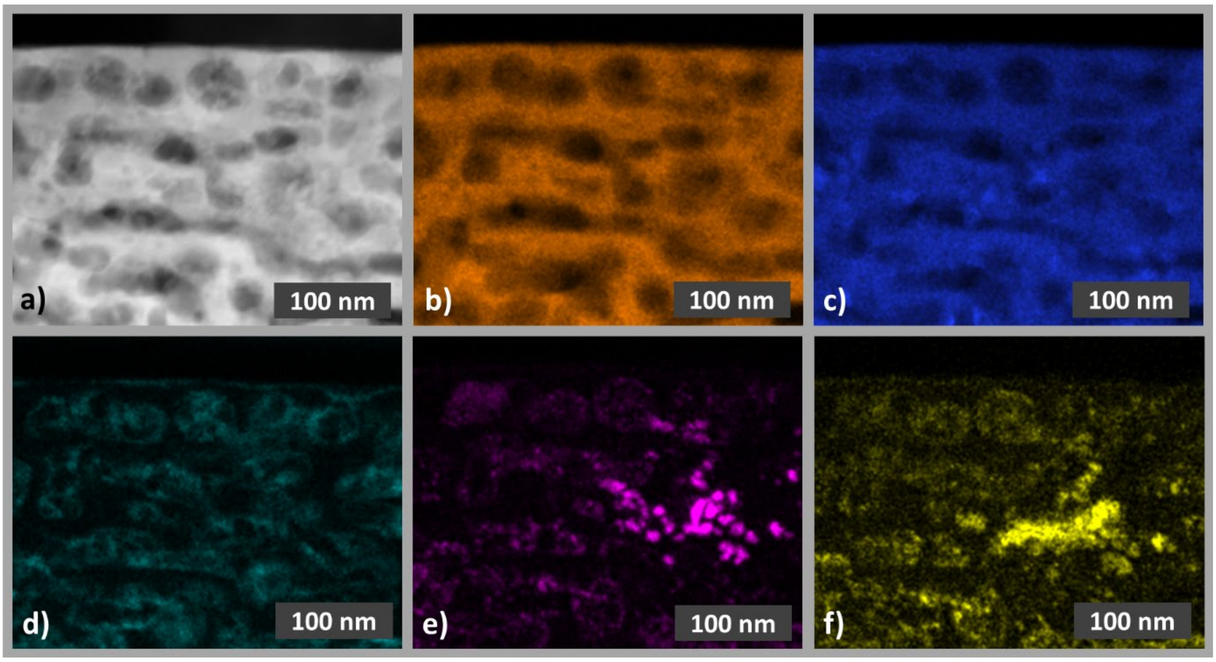

Figure S3: a) HAADF images of a lamella extracted from a *pristine* LTON photoanode. b) La, c) Ti, d) Ta, e) Ni, and f) Co elemental maps of the same cross sections (scale bar: 100nm)

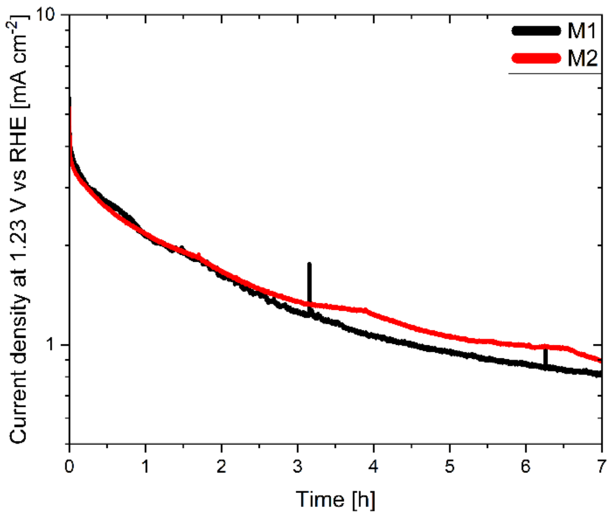

Figure S4: Chronoamperometries of two LTON-based photoanodes (M1 and M2) under an irradiance of 1 sun and at an electrolyte temperature of 34°C.

Table S1: Fitting parameters for fits of the chronoamperometries of two LTON based photoanodes under an irradiance of 1 sun and at an electrolyte temperature of 34°C (Equation 2).

	τ_{cp} [h]	$I_{0,cp}$ [mA cm^{-2}]	τ_{pc} [h]	$I_{0,pc}$ [mA cm^{-2}]	Adj. R^2	Ret_{CA} [%]
M1	1.05	2.04	9.91	1.57	0.993	52.3
M2	0.88	1.67	9.88	1.80	0.993	48.9

Table S2: Fitting parameters for fits of the chronoamperometries on LTON photoanodes at different irradiances with two exponential functions (Equation 2).

Irradiance [suns]	τ_{cp} [h]	$I_{0,cp}$ [mA cm^{-2}]	τ_{pc} [h]	$I_{0,pc}$ [mA cm^{-2}]	Adj. R^2
1	0.86	1.67	9.73	1.82	0.992
12	0.21	3.22	5.13	1.68	0.969
43	0.11	7.20	3.81	2.49	0.963
76	0.07	8.91	2.35	2.54	0.965
119	0.10	12.12	2.13	3.21	0.957

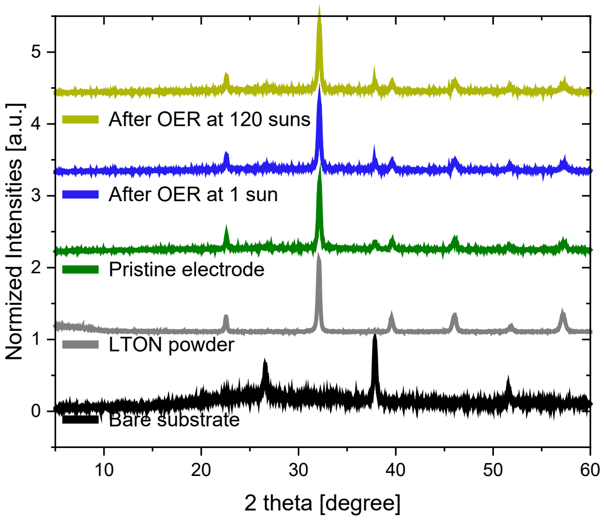

Figure S5: Normalised XRD Pattern of LTON based photoanodes before (green) and after seven-hour chronoamperometry under irradiances of 1 sun (blue) and 120 suns (yellow).

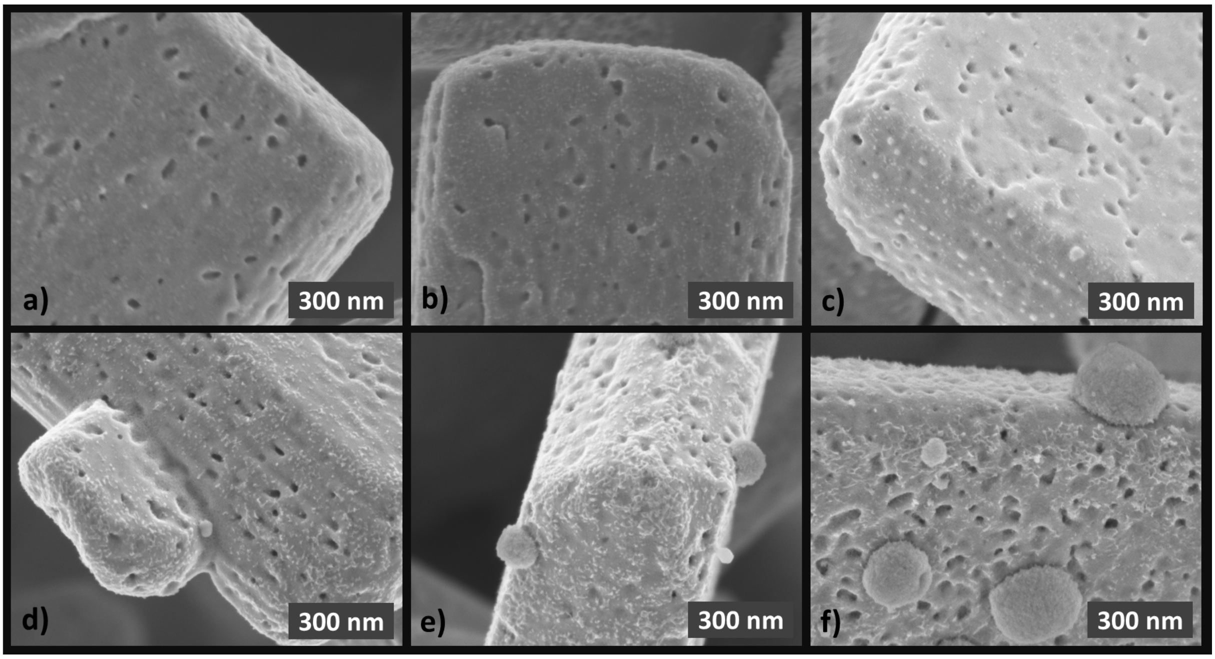

Figure S6: a) SEM image of a *pristine* LTON photoanode. SEM images of LTON photoanodes after seven-hour OER under irradiances of b) 1 sun, c) 12 suns, d) 43 suns, e) 76 suns and f) 120 suns (scale bar: 300 nm).

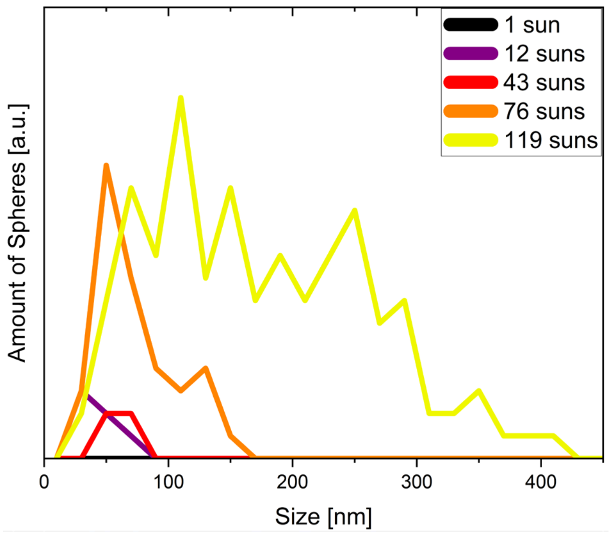

Figure S7: Quantity and size of the spheres on top of LTON photoanodes after seven-hour chronoamperometry at 1.23 V at different irradiances. For estimating the size distribution of the formed spheres at the different illumination intensities, the sizes of all spheres within a defined area (20.4 µm^2) were determined using the software ImageJ. Further the spheres were sorted into groups of similar sizes (i.e 0 – 20nm, 20 – 40 nm, etc.) and the number of spheres per group was plotted as function of the size for the different illumination intensities.

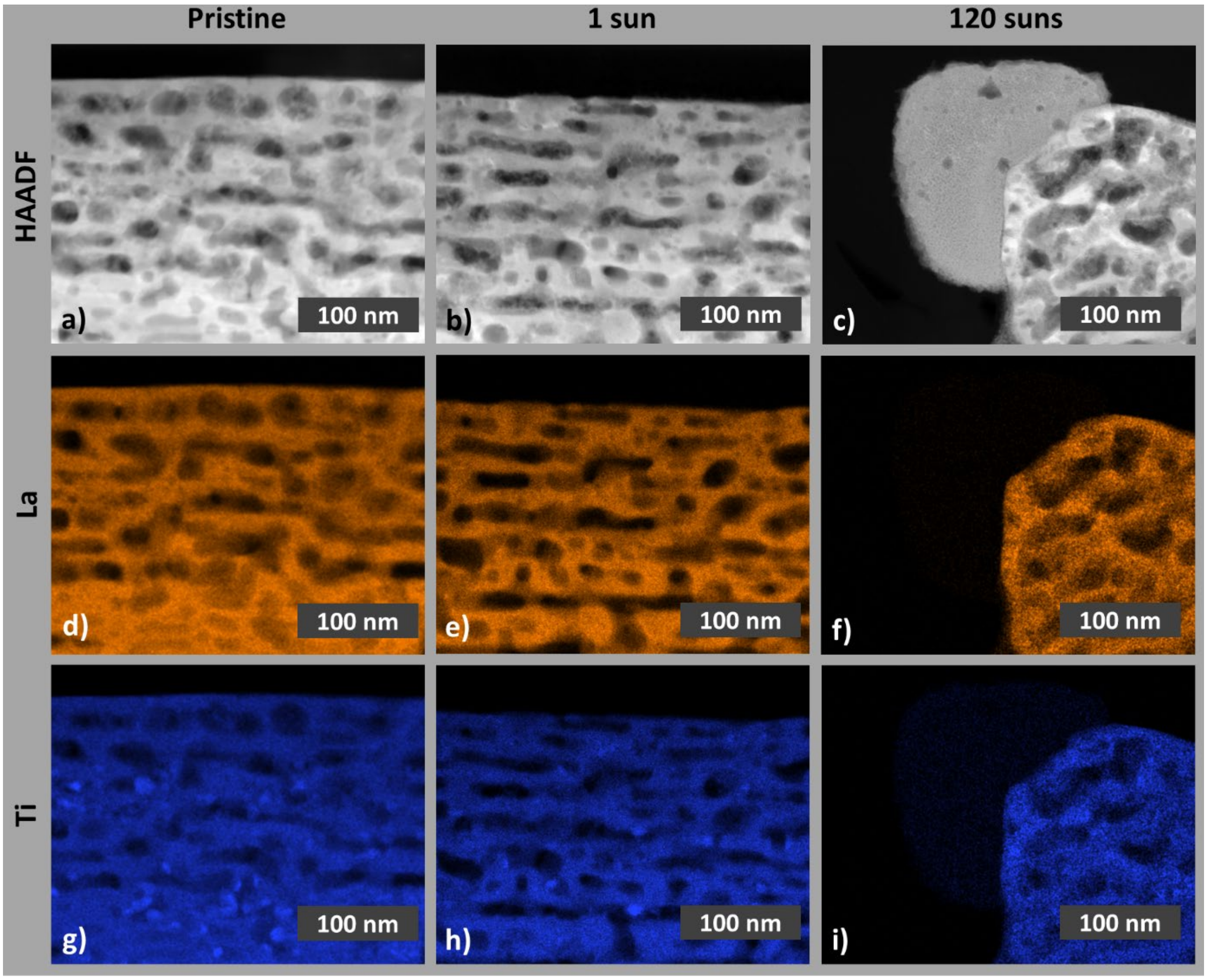

Figure S8: HAADF images of lamellae extracted from a) a *pristine* LTON photoanode, and LTON photoanodes after seven-hour of chronoamperometry under irradiances b) of 1 sun and c) 120 suns. d-e) La and g-h) Ti STEM-EDX elemental maps of the same cross sections (scale bar: 100nm).

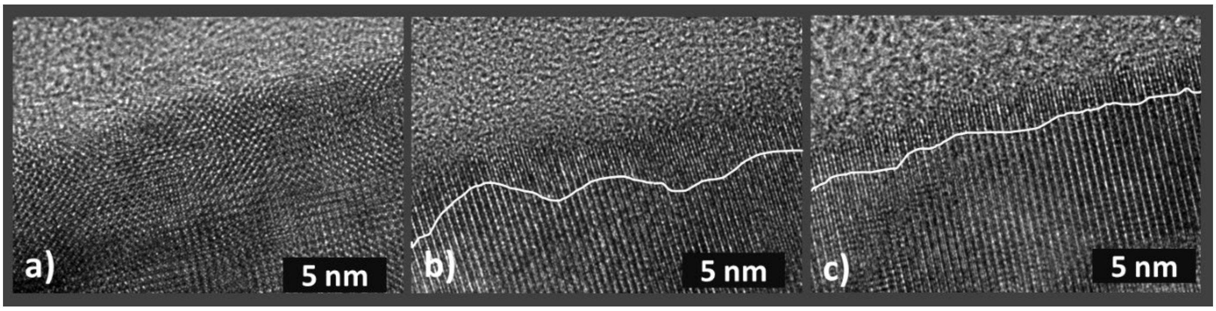

Figure S9: a) HREM image of a *pristine* LTON based photoanode and after seven-hour chronoamperometry at under irradiances of b) 1 and c) 120 suns. The white lines are guides to the eye to indicate the approximate location of the boundary between two regions with different crystal structures.

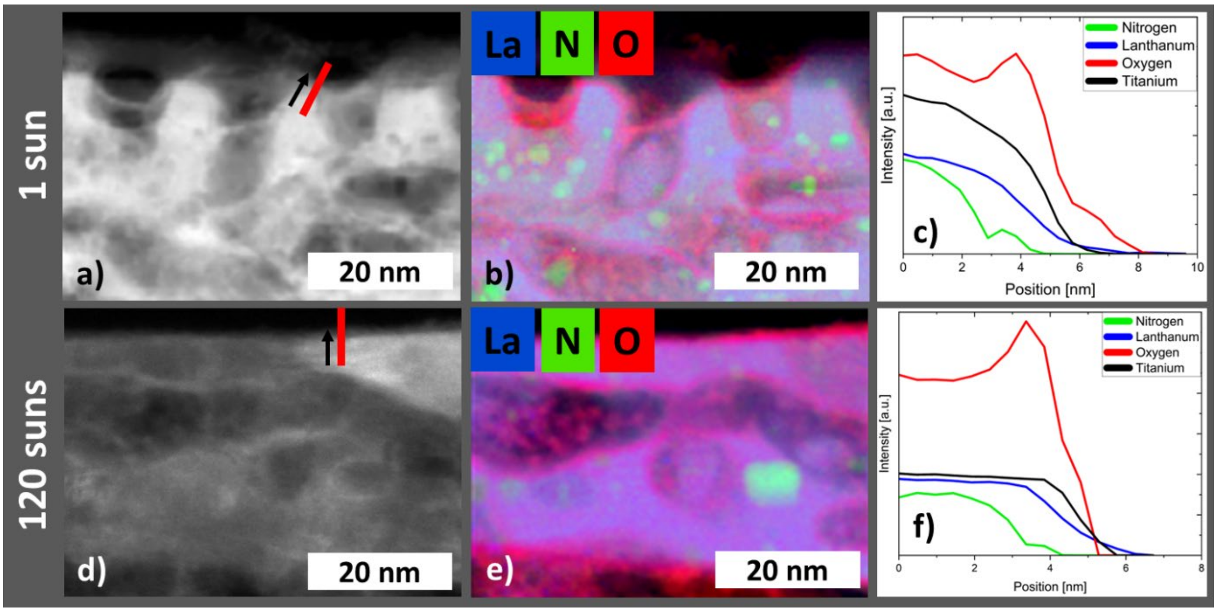

Figure S10: a) and d) ADF STEM images of lamellae extracted from LTON photoanode after seven-hour chronoamperometry under irradiances of 1 and 120 suns. b) and e) EELS composite maps of the La-M edge, O-K edge and the N-K edge of the same area. c) and f) Line profiles of the integrated La (turquoise), O (blue) Ti (black) and N (green) edge intensities (red lines in the corresponding ADF images indicate the location of the line profiles).

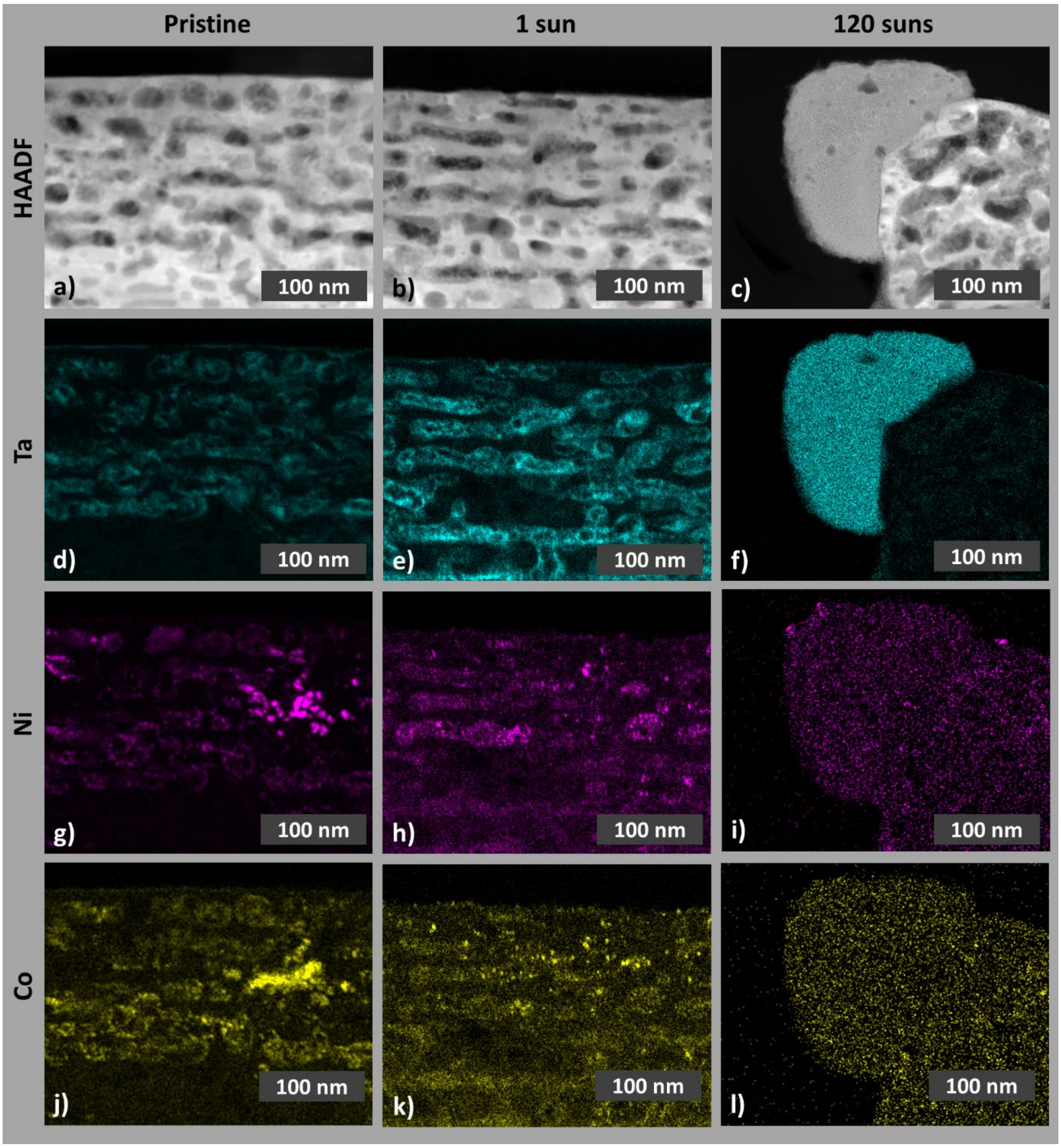

Figure S11: HAADF images of lamellae extracted from a) a *pristine* LTON-based photoanode, and LTON-based photoanodes after seven-hour chronoamperometry under an irradiance of b) 1 sun and c) 120 suns. d-f) Ta, g-i) Ni and j-l) Co, STEM-EDX elemental maps of the same cross sections (scale bar: 100nm)

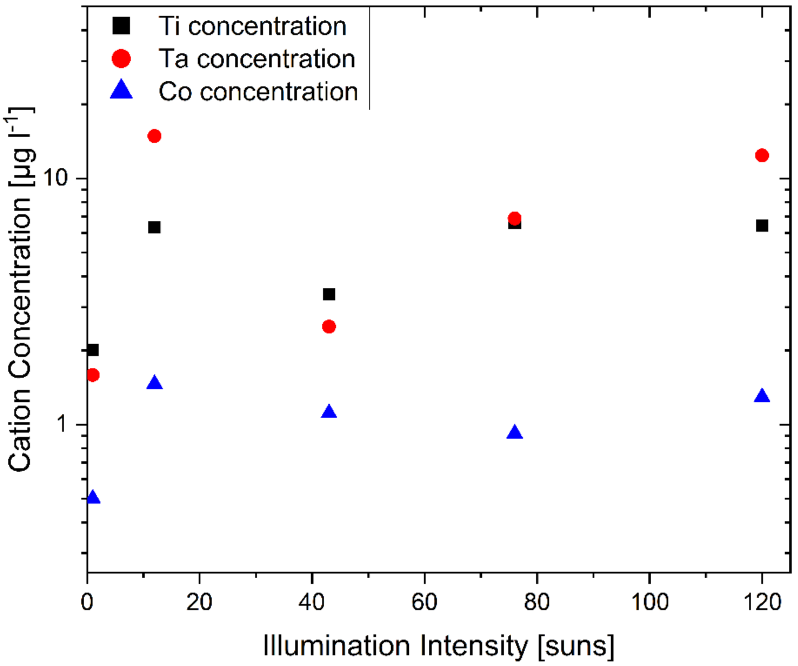

Figure S12: Concentrations of Ti, Ta and Co in the electrolyte after seven-hour chronoamperometries under different irradiances.

Table S3: Fitting parameters for fits of the chronoamperometries on LTON photoanodes at different electrolyte temperatures with two exponential functions (Equation 2).

**Electrolyte Temperature [°C]**	**τ_{cp} [h]**	**$I_{0,cp}$ [mA cm^{-2}]**	**τ_{pc} [h]**	**$I_{0,pc}$ [mA cm^{-2}]**	**Adj. R^2**
17	0.68	1.98	9.49	1.97	0.987
24	0.74	1.80	9.18	1.87	0.990
34	0.95	1.84	9.70	1.71	0.992
38	0.64	1.43	7.91	1.25	0.991
44	0.50	1.77	7.93	1.03	0.990
50	0.36	1.84	7.03	1.12	0.980

Table S4: Oxygen evolution rates of LTON particles at different electrolyte temperatures (Equation 1).

**Electrolyte Temperature [°C]**	**Oxygen Evolution Rate [µmol h^{-1}]**
3.4	0.9 ± 0.35
10.3	3.5 ± 0.25
15.3	5.8 ± 0.26
23.3	10.2 ± 0.25
24.9	11.0 ± 0.45
30.7	13.6 ± 0.53
36.2	16.4 ± 0.69

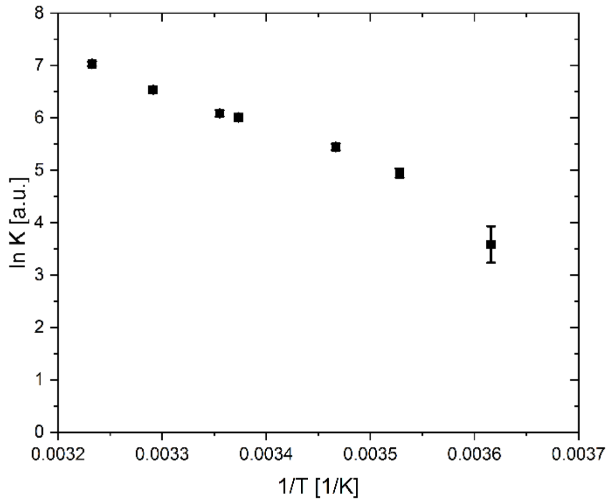

Figure S13: Arrhenius plot of the amount of oxygen evolved at different electrolyte temperatures (17°C – 50°C).

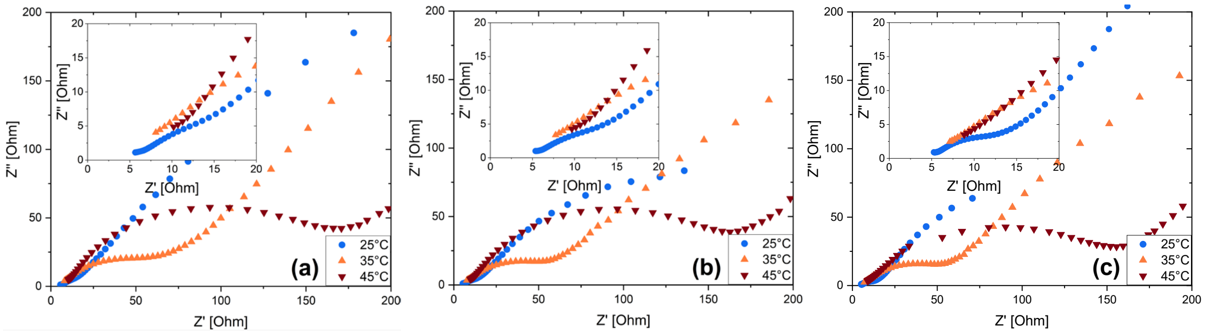

Figure S14: EIS measurements of LTON photoanodes at different electrolyte temperatures and under AMG 1.5 illumination at potentials of (a) 1.0 V vs RHE, (b) 1.2 V vs RHE and (c) 1.4 V vs RHE.

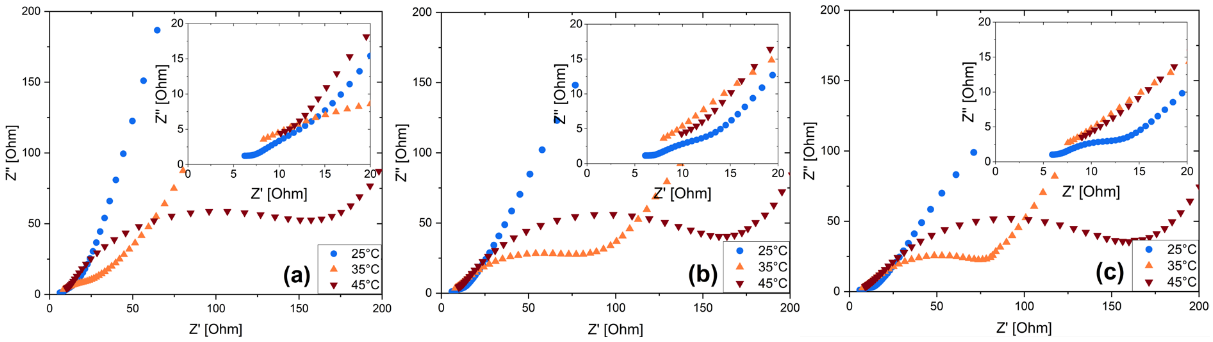

Figure S15: EIS measurements of LTON photoanodes at different electrolyte temperatures in the dark at potentials of (a) 1.0 V vs RHE, (b) 1.2 V vs RHE and (c) 1.4 V vs RHE

Table S5: Serial resistance of LTON based photoanodes at different electrolyte temperatures and applied potential, with and without illumination. The values were obtained by fitting of the Nyquist plots using the equivalent circuit displayed in Figure S1.

	Serial Resistance [Ohm]					
**Potential [V vs RHE]**	**25°C_dark**	**25°C_ill**	**37°C_dark**	**37°C_ill**	**45°C_dark**	**45°C_ill**
0.9	6.7	5.6	5.7	5.0	9.8	8.7
1.0	6.5	5.1	6.1	4.9	8.8	8.6
1.1	6.2	4.8	5.1	5.0	8.5	8.6
1.2	5.9	4.7	5.8	5.2	8.6	8.5
1.3	5.6	4.5	6.1	5.4	8.4	8.2
1.4	5.4	4.5	6.1	5.4	7.7	8.4

Table S6: Charge transport resistance of LTON based photoanodes at different electrolyte temperatures and applied potential, with and without illumination. The values were obtained by fitting of the Nyquist plots using the equivalent circuit displayed in Figure S1.

Charge Transport Resistance [Ohm]						
**Potential [V vs RHE]**	**25°C_dark**	**25°C_ill**	**37°C_dark**	**37°C_ill**	**45°C_dark**	**45°C_ill**
0.9	15.5	21.9	111.3	92.4	194.2	180.3
1.0	15.0	24.8	39.1	75.7	189.4	179.1
1.1	11.6	22.5	43.2	69.0	177.6	177.9
1.2	11.5	21.6	105.6	67.2	179.1	177.4
1.3	12.8	22.0	94.3	65.1	179.8	178.8
1.4	12.8	16.9	87.2	63.8	181.2	163.0

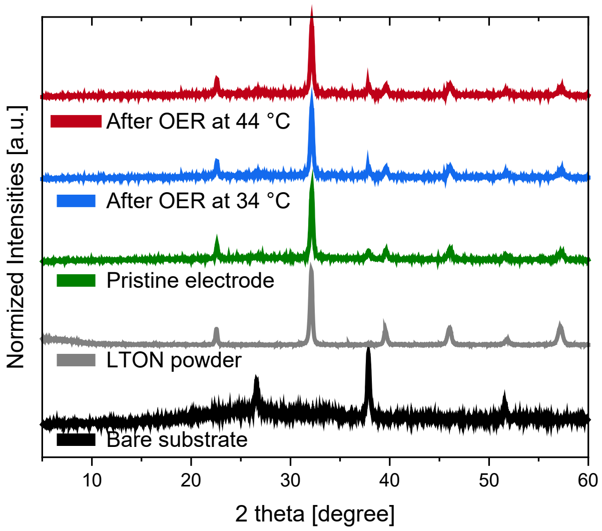

Figure S16: Normalised XRD Pattern of LTON based photoanodes before (green) and after seven-hour chronoamperometry at electrolyte temperatures of 34 °C (blue) and 44 °C (red).

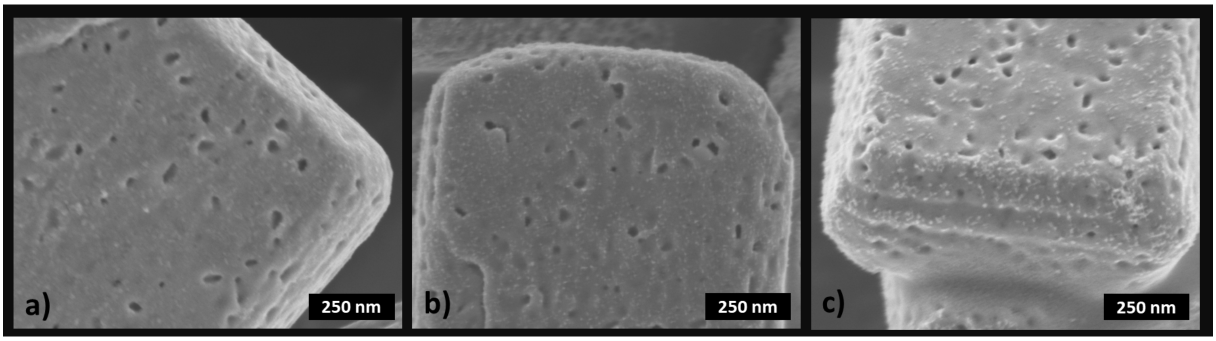

Figure S17: SEM images of a) a pristine LTON-based photoanode, and LTON-based photoanodes after seven-hour chronoamperometry with electrolyte temperatures of b) 34 °C and c) 44 °C (scale bar*:* 300 nm).

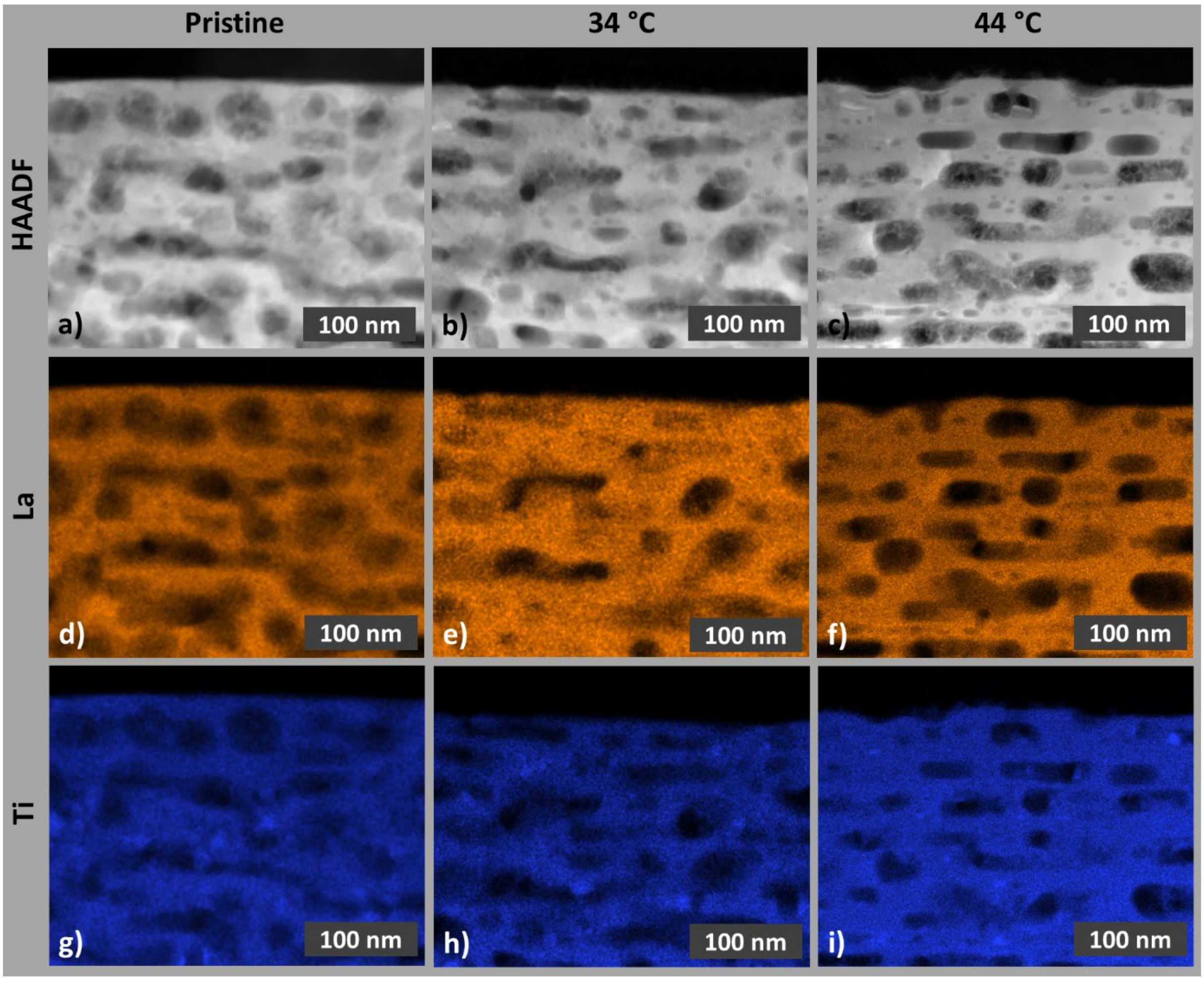

Figure S18: HAADF images of lamellae extracted from a) a *pristine* LTON photoanode, and LTON photoanodes after 7 h of chronoamperometry with electrolyte temperatures of b) 34 °C and d) 44 °C. d-f) La and g-i) Ti STEM-EDX elemental maps of the same cross sections (scale bar: 100nm).

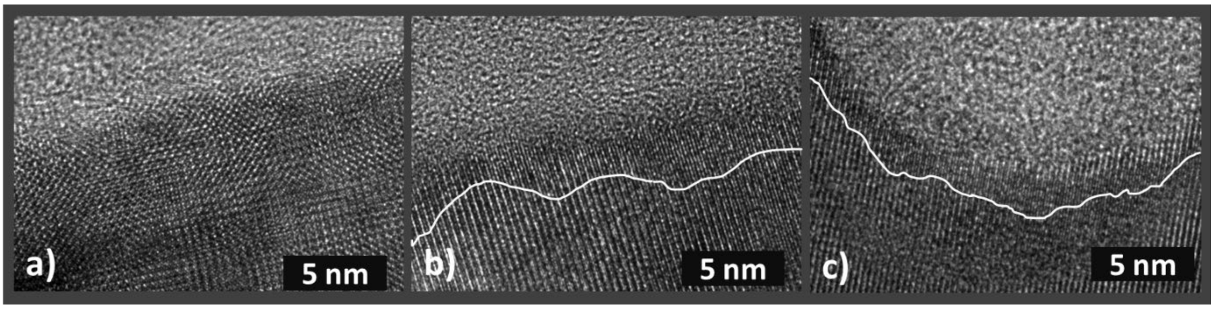

Figure S19: a) HREM image of a *pristine* LTON based photoanode and after seven-hour chronoamperometry at electrolyte temperatures of b) 34 °C and c) 44 °C. The lines serve as guide to the eye. The white lines are guides to the eye to indicate the approximate location of the boundary between two areas with different crystal structures.

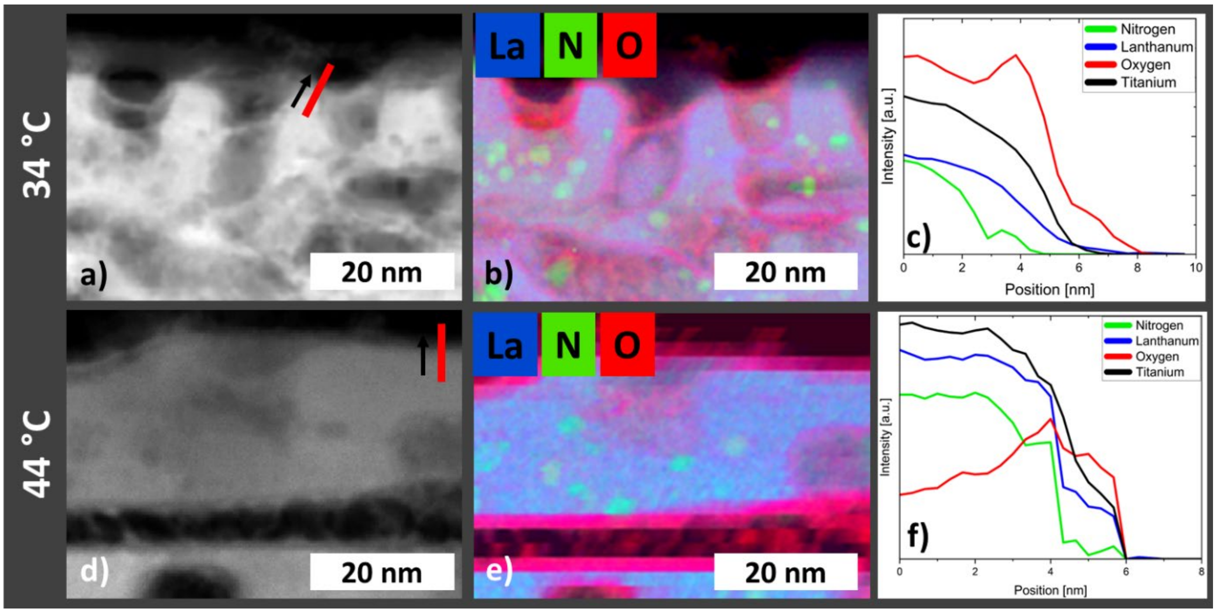

Figure S20: a) and d) ADF STEM images of a lamella extracted from LTON photoanode after seven-hour chronoamperometry with electrolyte temperatures of 34 and 44 °C b) and e) EELS composite maps of the La-M edge, O-K edge and the N-K edge of the same area. c) and f) Line profiles of the integrated La (turquoise), O (blue) Ti (black) and N (green) edge intensities (red lines in the corresponding ADF images indicate the location of the line profiles).

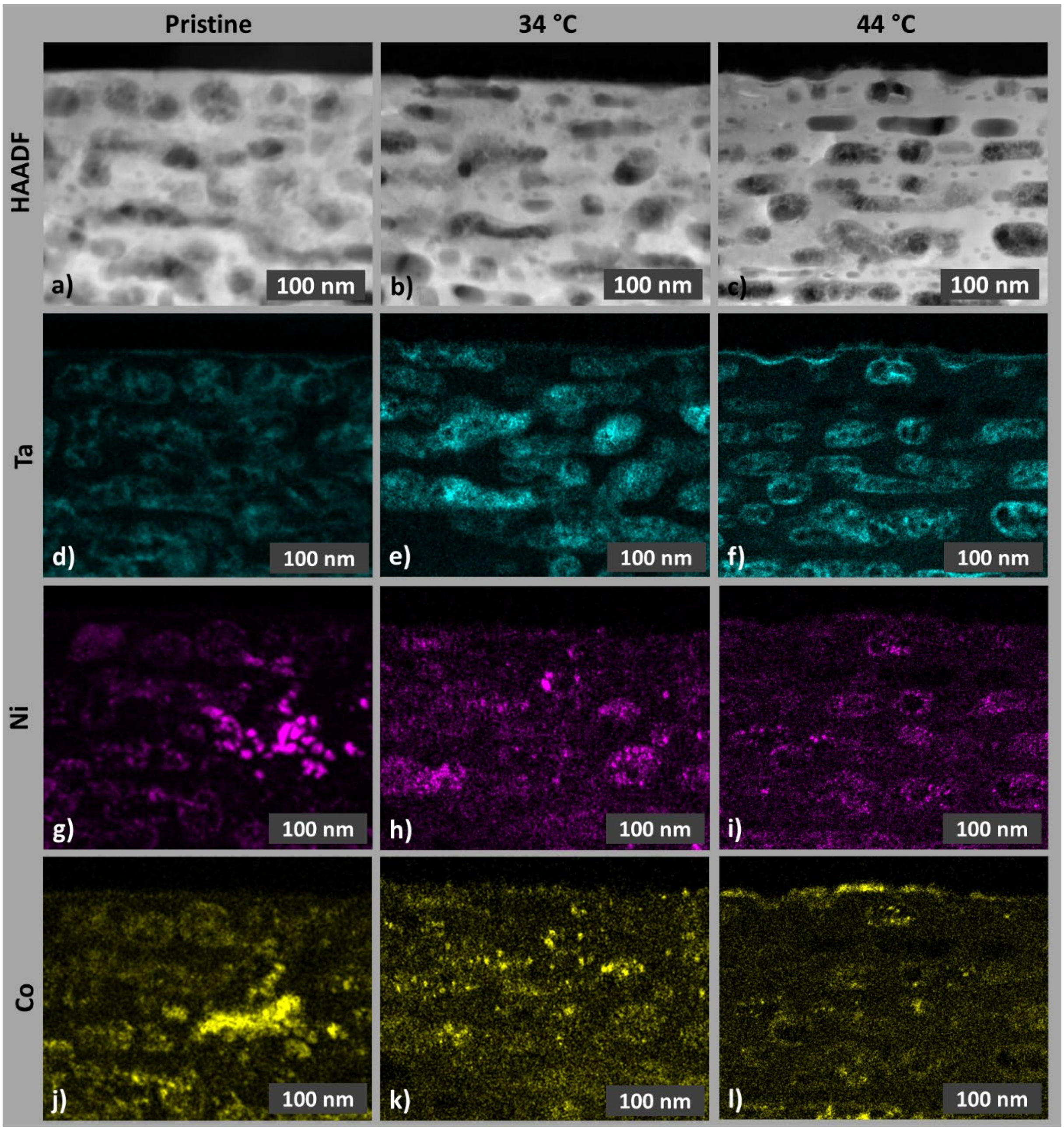

Figure S21: HAADF images of lamellae extracted from a) a *pristine* LTON-based photoanode, and LTON-based photoanodes after seven-hour chronoamperometry with electrolyte temperatures of b) 34 °C and c) 44 °C. d-f) Ta, g-i) Ni and j-l Co, STEM-EDX elemental maps of the same cross sections (scale bar: 100nm)

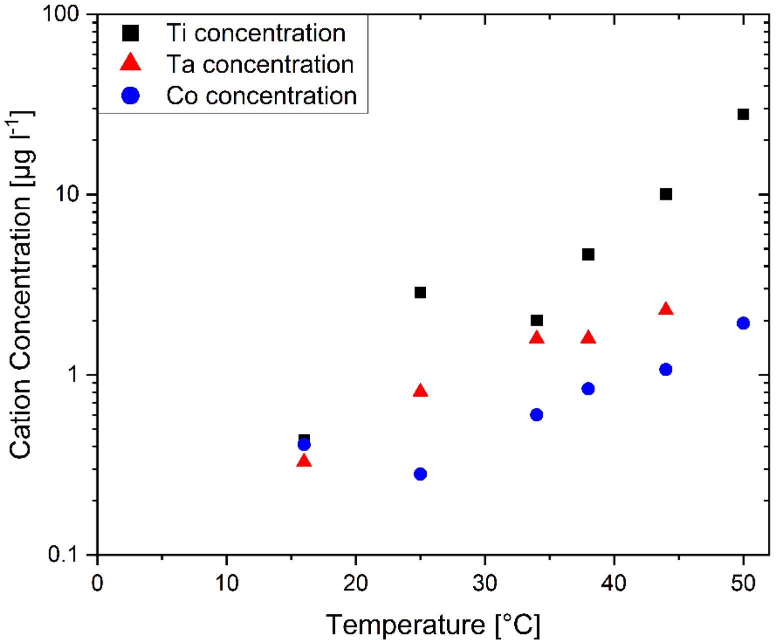

Figure S22: Concentrations of Ti, Ta and Co in the electrolyte after seven-hour chronoamperometries at different electrolyte temperatures.

1. Dias, P., et al., *Temperature effect on water splitting using a Si-doped hematite photoanode.* Journal of Power Sources, 2014. **272**: p. 567-580.
2. Klahr, B., et al., *Photoelectrochemical and impedance spectroscopic investigation of water oxidation with "Co-Pi"-coated hematite electrodes.* J Am Chem Soc, 2012. **134**(40): p. 16693-700.
